# Engaging the Concepts of Bimetallicity and Mechanical Strain for N_2_ Activation: A Computational Exploration

**DOI:** 10.1021/acsami.4c09691

**Published:** 2024-10-07

**Authors:** Omer Elmutasim, Louai Mahdi Maghrabi, Dattatray S. Dhawale, Kyriaki Polychronopoulou

**Affiliations:** † Department of Mechanical and Nuclear Engineering, Khalifa University of Science and Technology, Main Campus, P.O. Box 127788 Abu Dhabi, UAE; ‡ Center for Catalysis and Separations (CeCaS), Khalifa University of Science and Technology, Main Campus, P.O. Box 127788 Abu Dhabi, UAE; § CSIRO Energy, Private Bag 10, 3169 Clayton South, Victoria, Australia

**Keywords:** ammonia synthesis, Haber–Bosch reaction, nitrogen activation, density functional theory, alloying, bimetallic surfaces, mechanical strain

## Abstract

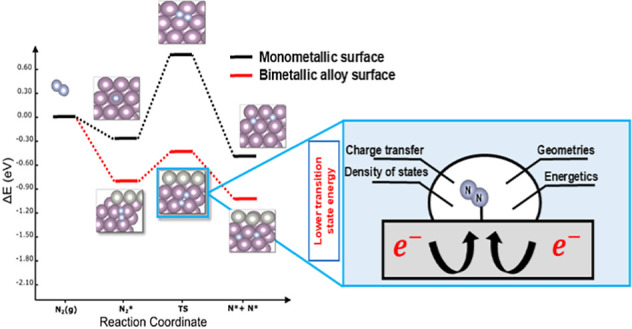

N_2_ activation is a vital step in the process toward NH_3_ production. NH_3_ synthesis has been considered a crucial process for the production of value-added chemicals and/or hydrogen carriers over recent years. In this work, density functional theory (ab initio) calculations are implemented for a thorough screening of bimetallic alloy surfaces using Fe, Ru, and Mo as the matrix (host) metals and Ag, Au, Co, Cu, Fe, Mo, Ni, Pd, Pt, Rh, and Rh as heterometals toward exploring the N_2_ catalytic activation (electronic and chemical characteristics); the monometallic surfaces are used for critical comparison in terms of their N_2_ activation behavior. In particular, adsorption geometries/energetics, density of states (DOS), and charge transfer are discussed. From the N_2_ activation on the surfaces, we could precisely capture the transition state of the N_2_ dissociation reaction/step. The effect of the metal alloying (geometrical and electronic factors) as well as the effect of applied mechanical strain, as a tuning factor of alloying, are both studied and thoroughly discussed. DOS studies revealed that the d-band center moved toward the negative direction for all late-TM-based alloys, thereby allowing the nitrogen molecule to adsorb weakly as compared to the early-TM surface alloys. In terms of the mechanical strain, for most of the alloy surfaces studied, apart from the Mo/Fe(110) one, the N_2_ binding energy varies as a linear function of the applied strain. The mechanical effect trend is in agreement with the charge transfer descending order followed: Fe/Mo(110) > Rh/Mo(110) > Au/Mo(110) > Pt/Mo(110) > Ni/Mo(110) > Ru/Mo(110) > Cu/Mo(110) > Ag/Mo(110) > Pd/Mo(110) > Au/Mo(110), pointing out that *Fe-functionalized Mo(110) surface presents the highest charge transfer of −2.14 |e| to the N*_2_*molecule*. This study aspires to provide navigation criteria through the abundant design criteria of N_2_ activation catalysts.

## 1 Introduction

The Haber–Bosch reaction is definitely one of the most important reactions of the 20th century with tremendous impact on significant, for mankind, sectors, such as fertilizers, chemical industry, medicine, biofuels, and ammonia production.^1^ The reaction itself was conceptualized in 1908 and put into large-scale production in 1913. The H–B process allows for large quantities of ammonia production; typically, 2500 tons of NH_3_ per day are produced in a single plant where natural gas and water are available in large quantities. The high emissions and the energy-intensive nature (temperature of 400–500 °C and pressure in the 150–250 atm range) of the H–B process seem to be the downside of this process. The commercial H–B process is carried out using a heterogeneous catalyst based on Fe promoted with Al_2_O_3_ and K_2_O. Though, N_2_ dissociation is the least favorable step over the Fe catalysts.^2^ The N–N triple bond is extremely stable (941 kJ/mol), which makes its activation and utilization difficult under ambient conditions,^1^ though the bond can be activated when electrons are transferred from the catalyst surface into the antibonding π-orbitals of N, facilitating the bond dissociation. To facilitate the transfer of electrons, the *N*_2_*-surface interaction should be optimized* (design of the active site). The active sites for N_2_ chemisorption can play the role of a pathway for the electron from the catalyst surface to N_2_ molecules. Extensive literature reports on the coordinatively unsaturated sites (CUS) and their important role in the events of N_2_ chemisorption.

It is now well accepted that the relative activity of metallic catalysts can be correlated to their binding energies with N-containing species in terms of a volcano-shaped relationship. Metals that bind nitrogen too strongly or too weakly are on either side of the volcano.^3^ For those metals having low binding energies, N_2_ dissociation is rate-limiting, whereas exhibiting high binding energies, facilitates N_2_ dissociation but desorption of the resulting atomic N is challenging. These correlations limit the rate of NH_3_ synthesis on transition metals to much lower values than would be possible on a material with both a low N_2_ dissociation barrier and more moderate intermediate binding energies.^3^ Clearly, an *important challenge is to break this type of scaling relationship*, which should lead to the development of catalysts yielding reaction rates that are potentially orders of magnitude higher than those of the current state of the art.

Thus, it is important to devise a *knowledge-based catalyst design* that could potentially replace the one used in the Haber–Bosch method while achieving high efficiency and energy saving toward sustainable NH_3_ production under ambient conditions.^3^ In this regard, the development of more efficient, low-cost, and environmentally friendly catalysts plays an important role in enhancing the reaction rate. The high stability of the N≡N triple bond makes N_2_ dissociation the most challenging NRR step, which is generally the potential-determining step.^4^ Therefore, to understand the catalytic characteristics and mechanism, it is important to study the N_2_ adsorption, activation, and dissociation.

The rationale of matrix metals chosen in this work is explained in what follows; the intensive search for an ideal active metal for ammonia production starts from nature. The biological fixation of N_2_ takes place through the nitrogenase enzyme, the major constituent of which is molybdenum (Mo) (active center). Therefore, Mo has been extensively used for designing NH_3_-producing catalysts.^5^ At the same time in the open literature, it is known that ruthenium (Ru) and its alloys are well-known as the most active catalysts for ammonia synthesis;^6^ however, the high cost associated with this noble metal is a major drawback. Therefore, there is a need for the development of inexpensive non-noble metal catalysts. Nørskov and co-workers predicted that the Co–Mo catalyst is more active than Fe- and Ru-based catalysts due to its suitable nitrogen binding energy.^7^ Density functional theory (DFT) calculations suggested that the nitrogen binding energy of pure Co is too weak and that of pure Mo is too strong, which results in a low ammonia synthesis activity. However, the nitrogen binding energy of the Co–Mo catalyst is intermediate between Co and Mo, positioned near the most active metal, Ru. Moreover, the utilization of multiple metals in and on the surface has been shown to be effective toward NH_3_ synthesis via unconventional electrochemical nitrogen and nitrogen oxide reduction using DFT computational studies. For example, Shi et al.^8^ found that doping g-C_3_N_4_ with two Mn atoms helped favor the synthesis of NH_3_. Shi et al.^9^ doped the Pt(110) surface with different 3d transition metals, where the screening study suggested favorable NO reduction catalysts upon the introduction Ti, Co, and Ni as single atom alloys. Lin et al.^10^ investigated single atom catalysts on monolayer BC_3_N_2_ that assisted in producing outstanding activity for electrochemical nitrogen reduction.

In addition, as elegantly explained in the work by Peterson and his group,^11^ one way to tailor the scaling relations is to modify the surface by straining it. Surface strain can be imposed either mechanically (loading) or by lattice mismatch (e.g., dealloying, core–shell nanoparticles, etc.). In the endeavor of designing catalysts for N_2_ activation, bridging of concepts of chemistry happening with strain is gaining traction. It has already been proved that mechanochemistry can tailor the activity of surface–adsorbate interactions for different catalytic reactions, such as DRM^12^ and methane oxidation.^13,14^

In this work, DFT calculations are used in order to screen the N_2_ activation capacity and the accompanied electronic interactions for a portfolio of bimetallic alloys based on Mo, Ru, and Fe metals, while using those three host/matrix metals *as reference ones*. The selection of the particular matrix metals (Mo, Ru, and Fe) has its routes into the biological fixation and industrial practicality as those explained above. Strategies for stimulating the N_2_ activation are thoroughly discussed.

## 2 Methodology

### 2.1 Computational Methods

DFT computations were carried out utilizing Quantum ESPRESSO package and projected augmented wave method.^15,16^ The computations were performed using the Perdew–Burke–Ernzerhof (PBE) exchange–correlation functional based on the generalized gradient approximation.^17^ The DFT-D3 method was employed for van der Waals dispersion corrections.^18^ Gaussian smearing with a width of 0.015 Ry was adopted to enhance the convergence. A plane wave expansion with a kinetic cutoff energy of 30 Ry was considered. Concerning structure relaxation, the convergence criteria for the total energy of self-consistent field iterations are set to 10^–4^ Ry and forces acting on each atom were converged 10^–3^ Ry/au. BFGS (Broyden–Fletcher–Goldfarb–Shanno) algorithm was adopted for geometry optimization. The spin-polarized computations were considered only for Fe(110), Co(0001), and Ni(111) and their bimetallic alloys. Partial density of states (PDOS) between nitrogen adsorbate and constituent metal atoms (in pure and alloyed surfaces) were calculated. Also, the d-band center was computed for both host and TM solute atoms on all bimetallic surfaces.

### 2.2 Computational Models

#### 2.2.1 Slab/Structures

From a thermodynamic perspective, the (110), (0001), and (111) facets are the *most stable surfaces* for *body-centered cubic (bcc) (Mo and Fe)*, hexagonal closed-packed *(hcp) (Ru and Co)*, and *face-centered cubic (fcc) (Ag, Rh, Ni, Au, Pt, Pd, and Cu) metals*, respectively.^19^ In regard to the bimetallic alloys, the crystal structures and lattice parameters of the host transition metals were adopted. Three atoms of the host metal were then replaced with solute metal atoms in the topmost layer, corresponding to 1/3 monolayer (ML) coverage. Five-layer slabs having a vacuum space of 15 Å were considered for all of the calculations. The three top layers and adsorbate were allowed to fully relax, while the two bottom layers were constrained at their bulk positions. The planar surfaces were modeled with 3 × 3 unit cells, while the Ru stepped surfaces were represented with 6 × 3 unit cells, wherein the three rows of Ru atoms in the topmost layer were removed to create a strip island having a width of three rows. The *K*-points grid of 3 × 3 × 1 and 3 × 6 × 1 were employed for flat and stepped surfaces, respectively. Considering isolated gas-phase computations, the N_2_ molecule was placed in a cubic simulation box having a dimension of 20 Å. Bader’s charge transfer analysis was performed to determine the charges of the surface and adsorbate as well as to provide charge density difference plots. Biaxial strain was isotropically applied by shrinking or stretching the simulation cell in both *x* and *y* axis. Selected values of biaxial strain in both compressive −3%, −1%, and tensile regime (1 and 3%) were applied.

#### 2.2.2 Formation Energy (*E*_f_) of Bimetallic Surfaces

To be convenient for practical applications, the catalytic surface must have durability and stability against deactivation induced by aggregation of the solute atoms. Thereby, the experimental feasibility of bimetallic surfaces was examined using the formation energy (*E*_f_). A more negative *E*_f_ implies higher probability for the formation of the binary alloy surface.^20^ The formula used for calculating the formation energy (*E*_f_) for bimetallic surfaces, such as in the case of the TM/Mo(110) bimetallic alloy, can be expressed as follows
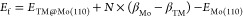
1where *N* represents the number of solute TM atoms and β_Mo_ and β_TM_ are the energy of atomic Mo in Mo(110) and TM in the corresponding TM slab, respectively.

#### 2.2.3 Adsorption Energy (*E*_ads_)

The adsorption energy (*E*_ads_) is determined from the difference between the total energy for the adsorbate–surface system (*E*_adsorbate–surface_) and those of the clean surface (*E*_surface_) and isolated gas phase adsorbate (*E*_adsorbate_) systems, as shown below
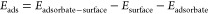
2

The transition states (TS) are obtained by climbing image nudge elastic band procedure. Convergence is reached when the total force of all images is less than 0.05 eV/Å. Six intermediate images were adopted for each CI-NEB calculation. The reaction energy (Δ*E*) and activation energy (*E*_a_) are calculated as follows
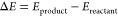
3
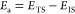
4where *E*_TS_ and *E*_IS_ are the energies of the transition and initial states, respectively.

#### 2.2.4 d-Band Center (ε_d_)

The d-band center ε_d_ was calculated as follows, where *E*_fermi_ is the Fermi level energy^21^
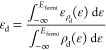
5

## 3 Results and Discussion

### 3.1 Stability of Bimetallic (Alloyed) Surfaces

Functionalization of the TM parent surface, via inclusion of guest transition-metal (TM) doped atoms, alters the electronic characteristics of the surface and thereby is anticipated to modify the catalytic performance. The *formation energy (E*_f_*)* for each of the bimetallic alloy surfaces, under study, namely, Fe-, Mo-, and Ru-based alloys, is listed in Table 1. Generally, TM-decorated Fe alloys presented relatively more negative formation energies compared with TM/Mo(110) and TM/st.Ru(0001) surfaces, which signifies that Fe-based alloys are highly stable. Among all studied Fe alloyed surfaces, **Cu/Fe(110)** surface exhibited the least formation energy (−12.51 eV). On the contrary, **Fe/Mo(110)** and **Fe/st.Ru(0001)** surfaces possess high *E*_f_, accounting for 0.15 and 0.06 eV, respectively, indicating the poor stability of those Fe-doped alloy surfaces (least likelihood to be formed). The introduction of Pd atom into Mo(110) and st.Ru(0001) resulted in the TM/Mo(110) and TM/st.Ru(0001) surfaces, which exhibited the most negative formation energies of −3.56 and −3.48 eV, respectively, among all Mo- and st.Ru-based alloy surfaces.

**Table 1:** Formation Energy (eV) of Various TM/**Mo**(110), TM/**Fe**(110), and TM/st.**Ru**(0001) Alloy Surfaces; TM Stands for Ag, Au, Co, Cu, Fe, Mo, Ni, Pd, Pt, Rh, and Ru

solute TM metal	formation energy (eV)		
	TM/Mo(110)	TM/Fe(110)	TM/st.Ru(0001)
Ag	–0.06	–0.43	–0.06
Au	–0.11	–0.21	–0.07
Co	–0.78	–9.96	–0.76
Cu	–0.80	–12.51	–0.87
Fe	0.15	-	0.06
Mo	-	–0.07	0.03
Ni	–0.09	–0.14	–0.05
Pd	–3.56	–3.57	–3.48
Pt	–0.21	–0.20	–0.08
Rh	–0.28	–1.19	–0.19
Ru	–0.10	–0.33	-

### 3.2 N_2_ Adsorption Calculations

The adsorption of N_2_ molecule on 12 (12) combinations of TM surfaces, such as Mo(110), Fe(110), Ni(111), Rh(111), Ag(111), Au(111), Pd(111), Pt(111), Cu(111), Co(0001), Ru(0001), and stepped Ru(0001) [hereafter named st.Ru(0001)] surface, and their binary alloys was thoroughly explored. The st.Ru(0001) substrate was investigated in terms of the influence of the presence of an extended edge of low-coordinated atoms on N_2_ activation. Here, N_2_ adsorption on monometallic and bimetallic surfaces is discussed. Detailed data of adsorption structures and energetics at different adsorption sites is presented in the Supporting Information section (Figures S2–S4 and Tables S1–S3, S6, and S7). Extensive discussion on the N_2_ adsorption over the monometallic surfaces is provided in the Supporting Information (Section S1). Monometallic surfaces are used as reference ones in the present study.

#### 3.2.1 N_2_ Adsorption on Bimetallic (Alloyed) Surfaces

##### 3.2.1.1 Geometry of Bimetallic Sites for Adsorption

**Figure 1:**
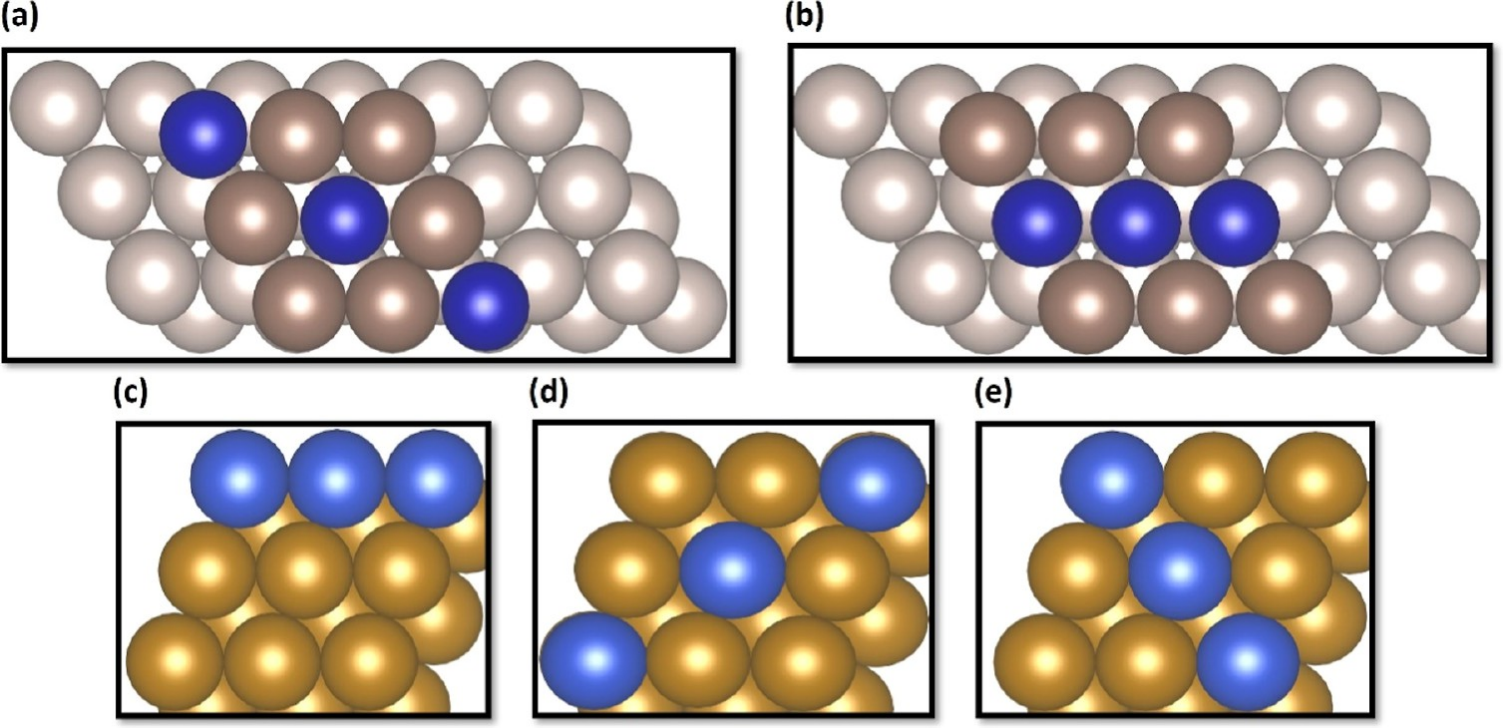
Top view of various patterns for the bimetallic alloy surfaces: (a) P1 and (b) P2 pattern of Co/stepped Ru(0001); (c) P3; (d) P4; and (e) P5 pattern of Cu/Fe(110). Ru: dark brown (topmost layer); Ru: light brown (2nd, 3rd, and 4th layer of the stepped surface); Co: dark blue; Fe: brown; and Cu: blue.

In bimetallic surfaces, if the solute metal prefers to stay in the bulk rather than on the surface, the influence of alloying is remarkably diminished owing to the reduction of the active sites associated with the guest element.^22,23^ Therefore, in this work, the bimetallic pairs that are not thermodynamically favorable for the solute metals to segregate toward the host surface are all ruled out. Based on Ruban et al. database,^24^ we have considered 30 bimetallic alloys with favorable surface segregation of the dopants among 110 binary metal alloy combinations composed of the 11 pure metals involved in this study. It should be highlighted that when a binary metal alloy surface is chosen, there could be a variety of surface patterns of alloy ordering. Herein, a solute coverage of 1/3 ML was selected as a ***model of surface alloy composition***. Two **surface alloy patterns (coded here as P1 and P2) for the hcp and fcc structures** and **three patterns (coded here as P3, P4, and P5) for the bcc surfaces** were considered, as shown in Figure 1a–e. The most stable surface arrangement was obtained using DFT computations for each binary alloy surface (tabulated in Tables S4 and S5, Supporting Information).

The bimetallic alloy surfaces possess some adsorption sites similar to the ones available on monometallic substrates but slightly different owing to the presence of solute atoms. For example, considering adsorption takes place on a Co/Ru(0001) surface having P1 pattern, there are various adsorption positions (depicted in Figure S1b, Supporting Information) based on the surrounding atoms, surface pattern of bimetallic alloy, and whether the adsorption occurs on terrace or step. As regards to the top site, the N_2_ molecule could be adsorbed atop the ruthenium (*t1 site*) or molybdenum (*t2 site*) atoms. In case of the adsorption on twofold bridge, there are two different sites; adsorption between two ruthenium atoms (b1) or between the ruthenium and molybdenum atoms (b2). For the adsorption on threefold hollow sites (hcp or fcc), there are only hcp and fcc hollow sites at the terrace; however, on the step, there are hybrid hollow sites (fcc or hcp), in which the N_2_ adsorbate is surrounded by two Ru atoms and one Mo atom. The N_2_ adsorption energies on various sites of the bimetallic surfaces are tabulated in Tables S6 and S7. Regarding the adsorption of atomic N, hollow sites are usually favorable thanks to their high coordination number.^25^

##### 3.2.1.2 N_2_ Activation on TM/Mo(110) Alloys

**Figure 2:**
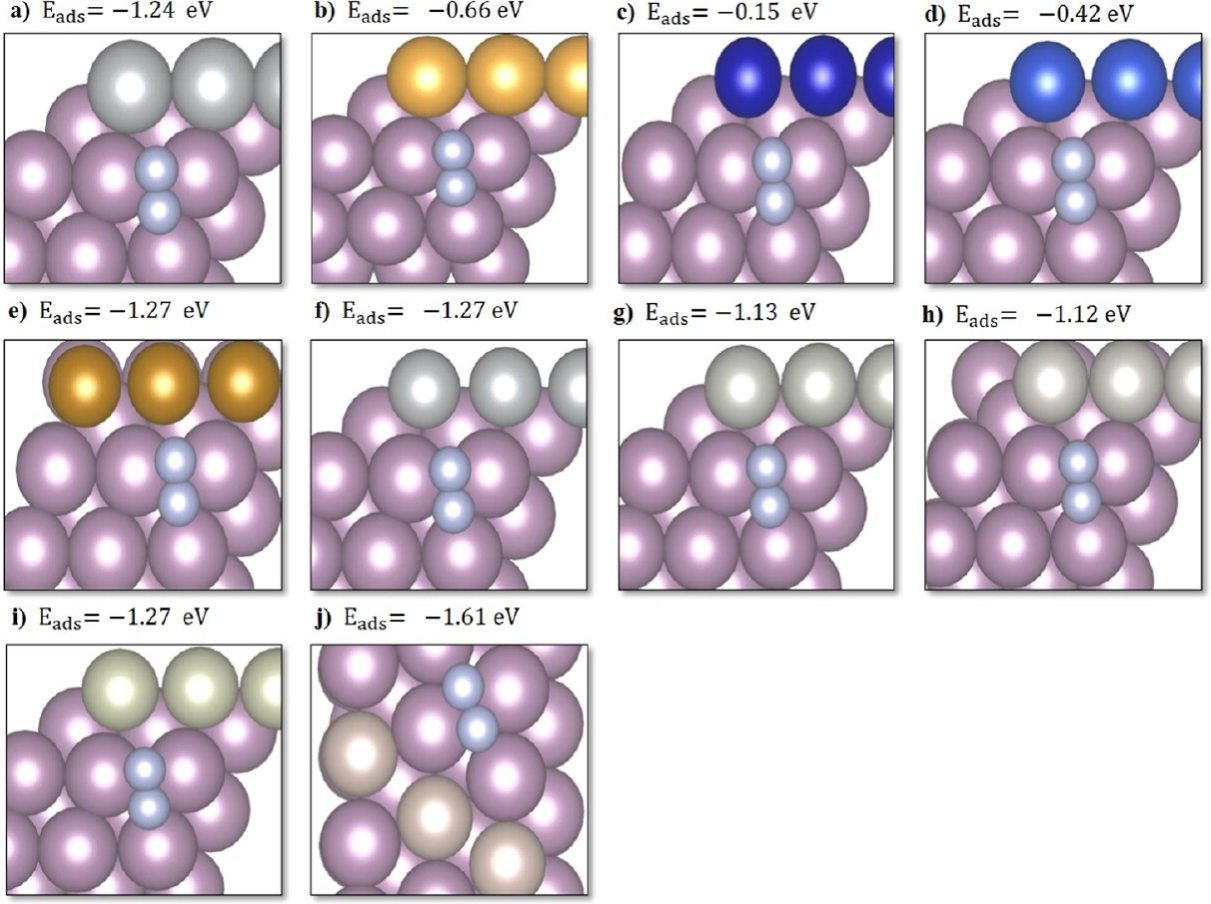
Top views of adsorbed N_2_ molecule on **Mo-based alloy** surfaces: (a) Ag/Mo(110); (b) Au/Mo(110); (c) Co/Mo(110); (d) Cu/Mo(110); (e) Fe/Mo(110); (f) Ni/Mo(110); (g) Pd/Mo(110); (h) Pt/Mo(110); (i) Rh/Mo(110); and (j) Ru/Mo(110). (N: light blue; Ag: light gray; Au: yellow, Co: dark blue; Cu: blue; Fe: brown; Mo: purple; Ni: light gray; Pd: dark gray; Pt: gray; Rh: gold; and Ru: light brown).

The first step of ammonia synthesis process is the adsorption of a nitrogen (N_2_) molecule on the catalytic surface, and its initial adsorption conformation plays a key role in the subsequent reaction route.^26^ Considering the cases of TM/Mo(110) bimetallic alloys, all possible adsorption configurations at various sites were investigated and the chemisorption state of N_2_ was studied for all studied Mo-based alloys. The most stable binding conformation of N_2_ for each alloy surface with its corresponding adsorption energy (*E*_ads_) is depicted in Figure 2 and the structural parameters are tabulated in Table 2. The N_2_ chemisorption energies, varied from −1.12 to −1.61 eV, follow a strength sequence of Ru/Mo(110) > Cu/Mo(110) > Co/Mo(110) > Ni/Mo(110) = Fe/Mo(110) = Rh/Mo(110) > Au/Mo(110) = Pd/Mo(110) > Pt/Mo(110) = Ag/Mo(110). It can be noted that the N_2_ binds most favorably on the **Ru/Mo(110)** alloy with an adsorption energy of −1.61 eV, implying a strong interaction between the N_2_ molecule and the involved surface. Compared to the bare Mo(110) surface, the dispersion of Ru atoms on Mo(110) surface results in a stronger chemisorption. The adsorption geometries possess a parallel orientation with distances [*d*_(surf–ads)_] of 1.94–2.17 Å above the surfaces, while the N–N bond lengths [*d*_(N–N)_] of 1.28–1.39 Å was found (N–N gas phase length 1.19 Å). It is noteworthy that the threefold hollow site is the most favorable adsorption site for N_2_ on all TM/Mo(110) alloys, thanks to its high coordination number. Although N_2_ cannot be stabilized on some pure surfaces, such as Ag(111), Au(111), and Cu(111), the introduction of these atoms into the topmost layer of Mo(110) has considerably enhanced their stability for N_2_ chemisorption. This result has design implications for the engineering of the active site for N_2_ adsorption.

**Table 2:** Structural Parameters of the Most Stable Geometries of N_2_ Molecule and Adsorption Energy (*E*_ads_) on Various TM/Mo(110), TM/Fe(110), and TM/st.Ru(0001) Alloy Surfaces

solute TM metal	*d*_(N–N)_ (Å)			*d*_(surf–ads)_ (Å)			*E*_ads_ (eV)		
	TM/Mo(110)	TM/Fe(110)	TM/st.Ru(0001)	TM/Mo(110)	TM/Fe(110)	TM/st.Ru(0001)	TM/Mo(110)	TM/Fe(110)	TM/st.Ru(0001)
Ag	1.29	1.13	1.29	2.15	1.92	2.03	–1.12	–1.57	–0.35
Au	1.28	1.23	1.30	2.14	1.99	2.02	–1.13	–0.55	–0.18
Co	1.29	1.26	1.33	2.14	1.92	1.92	–1.34	–1.57	–1.00
Cu	1.30	1.26	1.29	2.12	1.90	2.05	–1.57	–1.59	–0.62
Fe	1.29	-	1.30	2.17	-	2.05	–1.27	-	–0.50
Mo	-	1.31	1.33	-	1.98	2.03	-	–1.98	–1.25
Ni	1.29	1.28	1.31	1.98	1.99	1.94	–1.27	–1.23	–0.94
Pd	1.29	1.13	1.30	1.96	1.87	2.03	–1.13	–1.02	–0.62
Pt	1.29	1.25	1.31	1.97	1.20	2.05	–1.12	–1.10	–0.60
Rh	1.29	1.29	1.31	1.94	1.20	2.04	–1.27	–1.20	–0.90
Ru	1.39	1.26	-	2.08	1.86	-	–1.61	–1.92	-

##### 3.2.1.3 N_2_ Activation on TM/Fe(110) Alloys

In terms of TM/Fe(110) bimetallic alloys, the most stable configurations for chemisorbed N_2_ along with its corresponding *E*_ads_ are shown in Figure S5. The molecular axis of N_2_ aligned parallel to all TM/Fe(110) alloys, except for Ag/Fe(110) and Pd/Fe(110); wherein perpendicular orientation of N_2_ adsorbate, coordinated to the Fe-top site, was observed. The structural parameters of N_2_ are considerably different from those of the free N_2_ molecule, as listed in Table 2. It was found that the most preferable binding site of N_2_ on TM/Fe(110) (TM = Co, Mo, and Ru) surfaces features at least one N atom coordinated to a hybrid hollow site, formed by TM and Fe atoms. At these adsorption positions, the *d*_(N–N)_ bond length stretched from 1.11 Å, in the gas phase state, to 1.26 Å (Co and Ru) and 1.31 Å (Mo), signifying that N_2_ was fully activated. On the other hand, alloying the Fe(110) surface with TM (=Au, Cu, Ni, Pd, Pt, and Rh) exhibits a preference for N_2_ binding with Fe atoms only instead of TM atoms. Overall, apart from TM/Fe(110) (TM = Ni, Cu, and Rh), the degree of N_2_ activation is a bit lower than those obtained on hybrid sites, as confirmed by the slight elongation of the N–N bond on these surfaces in the range of 1.13–1.25 Å. The N_2_ adsorption strength was followed in the descending order: Mo/Fe(110) > Ru/Fe(110) > Cu/Fe(110) > Ag/Fe(110) = Co/Fe(110) > Ni/Fe(110) > Rh/Fe(110) > Pt/Fe(110) > Pd/Fe(110) > Au/Fe(110), pointing out that Mo-decorated Fe(110) surface presents the strongest interaction with N_2_. Because Mo(110) and Fe(110) possess the highest reactivity toward N_2_, as previously mentioned in Section 3.2.1, a strongly bound motif was obtained for Mo/Fe(110), whereby the N≡N bond is most likely to be cleaved [*d*_(N–N)_ = 1.31 Å], rendering the progression of subsequent reaction steps of ammonia synthesis process easier.

##### 3.2.1.4 N_2_ Activation on TM/st.Ru(0001) Alloys

Previous theoretical and experimental findings have revealed that low-coordinated adsorption positions exhibit higher catalytic activity for activation of various small molecules and could influence the surface chemistry.^27,28^ Controlled step decoration with TM (=Co, Mo, Fe, Ni, Rh, Ag, Au, Pd, Pt, and Cu) atoms was employed to locally tune the reactivity of st.Ru(0001) step sites. This particular method of modifying st.Ru(0001) features a new approach to catalyst design where the step atoms are altered, wherein one atom of the step is decorated with a TM atom to discriminately tune the binding of a particular adsorbate, thereby changing the reaction mechanism occurring on the substrate. Adsorption geometries and structural parameters of N_2_ molecules in their lowest energy configurations on the stepped TM/st.Ru(0001) alloys are presented in Figure S6 and Table 2, respectively. On all TM/st.Ru(0001) substrates, there is preference by N_2_ for the fivefold site, wherein one N binds on the step edge atom and the second N inclined to the lower terrace, with a TM–N distance [*d*_(surf–ads)_] range of 1.92–2.05 Å and N–N bond lengths of 1.29–1.33 Å. The adsorption preference of N_2_ on stepped Ru(0001) was also found in previous work.^29^ It should be highlighted that introducing TM atoms into st.Ru(0001) surface, i.e., *modifying the atomic composition of the step edge, regulates the electronic structure of Ru and promotes the catalytic reactivity*. For instance, doping st.Ru(0001) with all TM-doped atoms studied, except for Mo, has led to reduction in the binding energy of atomic N from −0.90 eV, on the parent st.Ru(0001) surface, to lower values ranging from 0.1 to −0.87 eV on the TM-decorated st.Ru(0001) surfaces (Table S11). Moderate binding between the N atoms and st.Ru-based alloy surfaces is beneficial, especially given the high activity of the Ru catalysts for ammonia synthesis reaction. The binding is neither weak to impair the adsorption of atomic N on Ru surface nor too strong to make the desorption difficult; so it is clear that a great trade-off is achieved by proper selection of the alloying heteroatoms.^20^ It is clear that the N_2_ species show a distinct chemisorption behavior on the binary alloy surfaces, compared to the case of the parent TM surfaces, for both the binding conformation and the relative stability.

### 3.3 N_2_ Dissociation on Metal Alloy Surfaces

#### 3.3.1 N_2_ Dissociation on TM/Mo(110) Alloys

**Figure 3:**
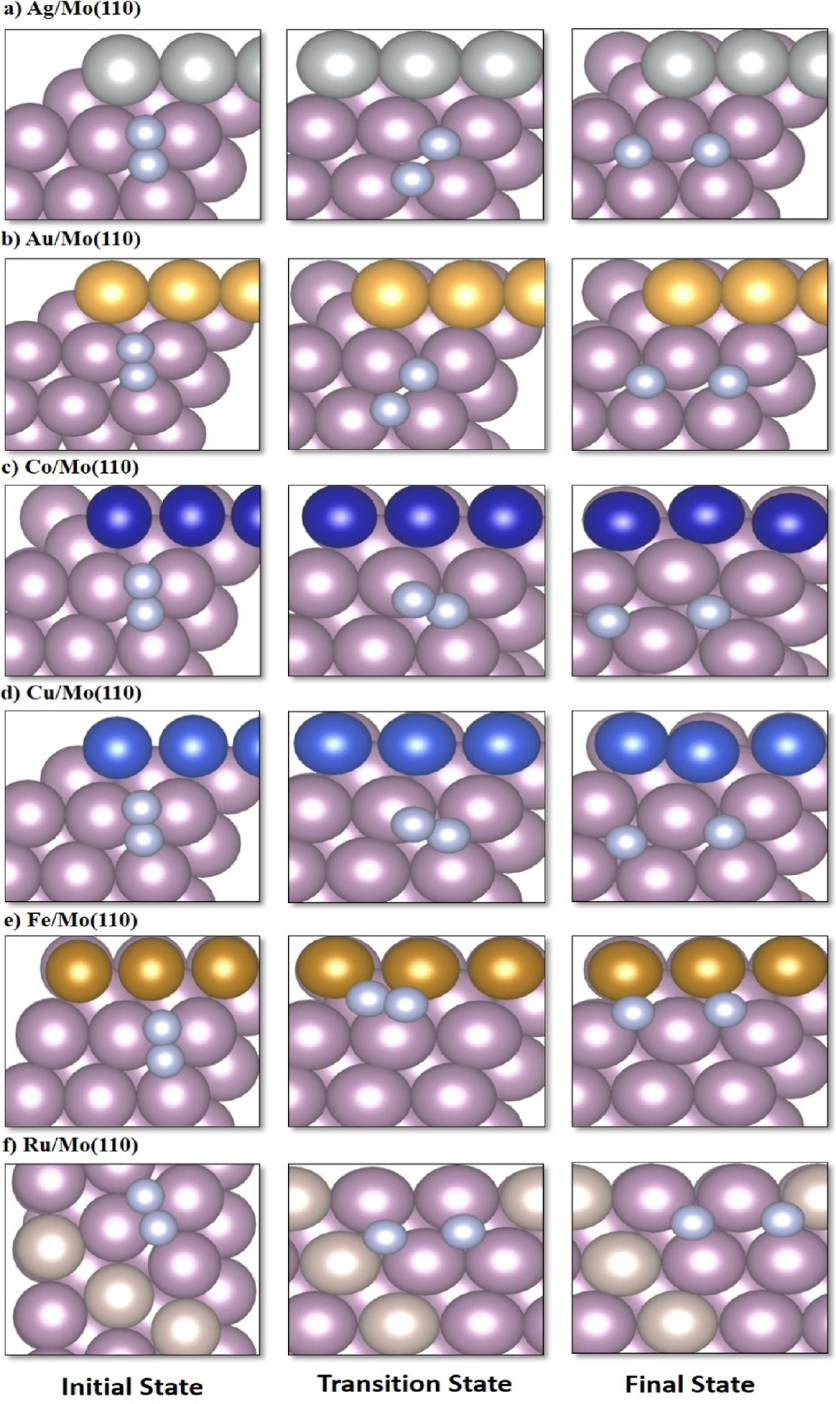
Top views of initial (IS), transition (TS), and final states (FS) of N_2_ dissociation on **Mo-based alloys**: (a) Ag/Mo(110); (b) Au/Mo(110); (c) Co/Mo(110); (d) Cu/Mo(110); (e) Fe/Mo(110); and (f) Ru/Mo(110) surfaces.

**Figure 4:**
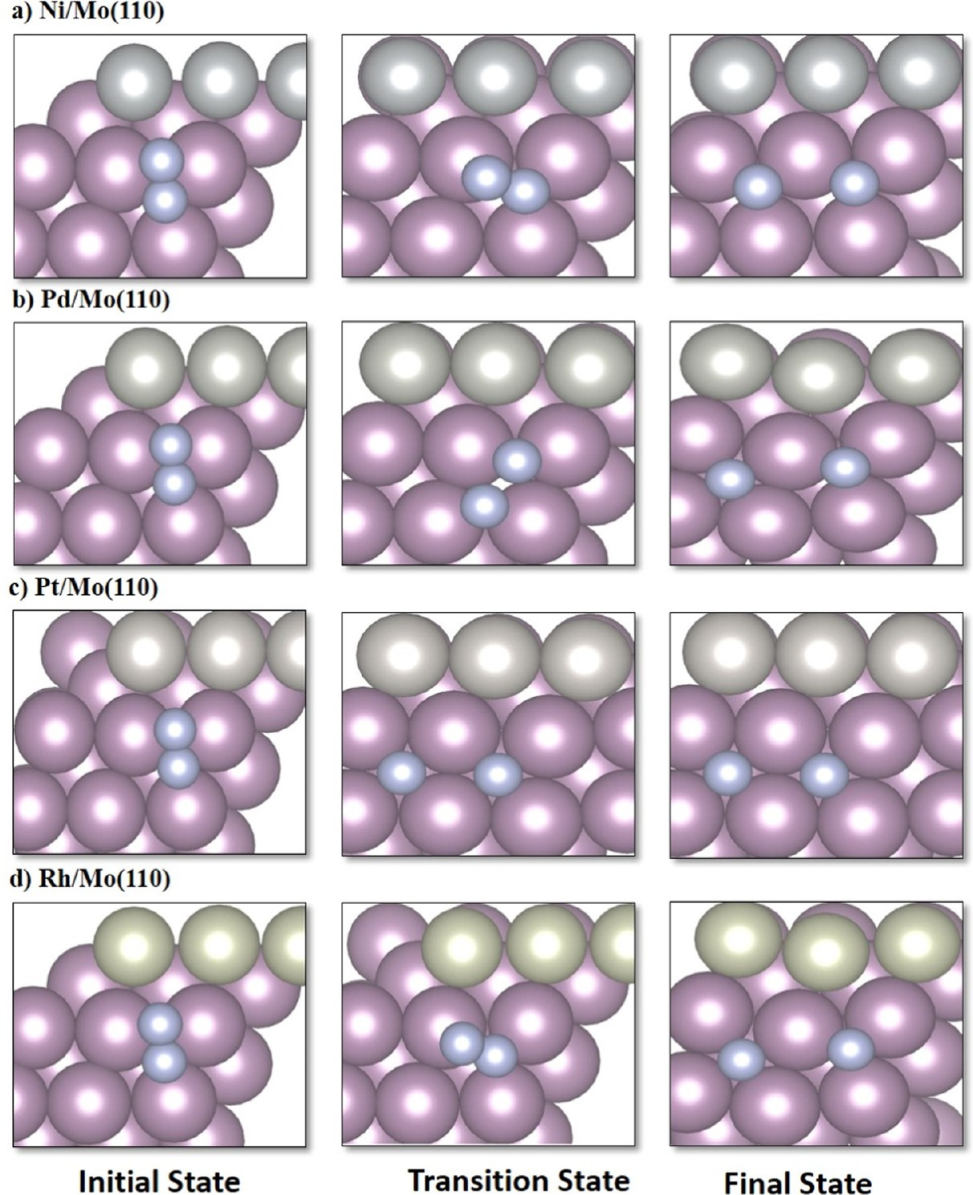
Top views of initial state (IS), transition state (TS), and final state (FS) of N_2_ dissociation on **Mo-based alloys**: (a) Ni/Mo(110); (b) Pd/Mo(110); (c) Pt/Mo(110); and (d) Rh/Mo(110) surfaces.

**Figure 5:**
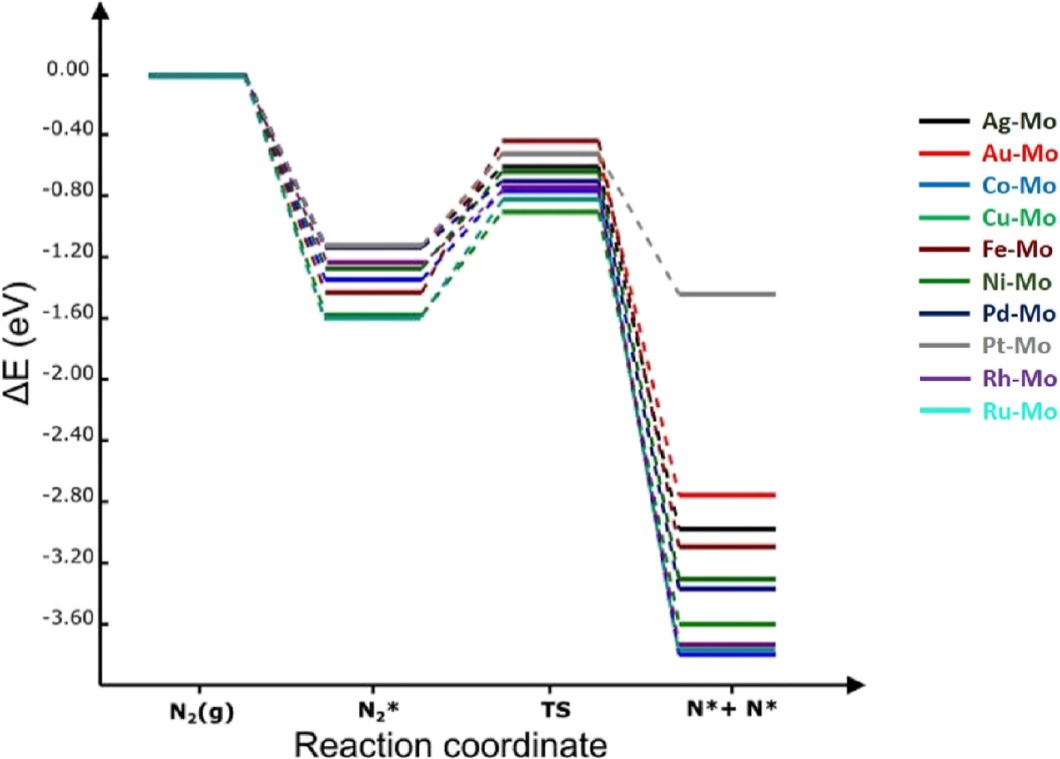
Potential energy landscape of N_2_ dissociation process on **Mo-based alloy** surfaces at 0 K. The energies are computed with respect to the N_2(g)_ molecule.

Jacobsen et al.^30^ postulated that the interaction energy between nitrogen adsorbate and mixed site of the bimetallic substrate is simply an intermediate between that of the individual metal components. Thereby, combining metals with strong and weak adsorption energies of nitrogen is anticipated to result in the desired intermediate adsorption strength that is beneficial for lowering the dissociation barrier and thereby resulting in high reactivity, as bolstered by the volcano-shaped relation between N_2_ adsorption energy and NH_3_ synthesis activity of various catalysts. According to the *volcano-shaped relation*, they have anticipated that combining Mo (which adsorbs N too strongly) and Co (which adsorbs N too weakly) should result in an enhanced performance. This was exactly what was found experimentally.^30^ A Co–Mo catalyst was designed under this principle and exhibited NH_3_ synthesis activity much higher than that of individual components and even much better than Ru and Fe catalysts. Similarly, alloying Mo(110) surface, possessing the strongest adsorption energy of N_2_ among all monometallic surfaces except for Fe(110), with other TM atoms is anticipated to reduce the kinetic barrier for N–N bond cleavage than that of the constituents. In this context, the dissociation reaction of N_2_ was explored on TM/Mo(110) alloys and the reaction pathway, encompassing the initial state (IS), transition state (TS), and final state (FS) structures, on each Mo-based alloy surface is shown in Figures 3 and 4. The corresponding potential energy landscape is shown in Figure 5. The process of breaking N–N bonds on Mo-based alloy surfaces can be divided into two types; first, for TM/Mo(110) (TM = Pd, Rh, Ag, Pt, Au, Co, Ni, and Cu), the N_2_ molecule is positioned on the hollow site of the IS structure. The two nitrogen atoms of the FS structure are situated on the hollow site. Second, for TM/Mo(110) (TM = Ru and Fe), the IS structure is similar to the previous type, whereas the dissociated nitrogen atoms of the FS structure are adsorbed on the hybrid hollow site, formed by one TM (TM = Ru and Fe) atom and two Mo atoms. The dissociation barrier increased in the following order on TM/Mo(110) alloys: Pd (0.43 eV) < Rh (0.51 eV) < Ag (0.52 eV) < Co (0.58 eV) < Pt (0.60 eV) < Au (0.61 eV) < Ni (0.64 eV) < Cu (0.67 eV) < Ru (0.77 eV) < Fe (1.00 eV). It is noteworthy that all surfaces, apart from TM/Mo(110) (TM = Ru and Fe) surfaces, presented energy barriers lower than that of the bare Mo(110) surface. The highest activation energy observed on Fe/Mo(110) surface, as compared to the pristine Mo(110), could be ascribed to the higher strength of atomic N binding energy on both individual Fe(110) and Mo(110) surfaces, in accordance with volcano-shaped relation reported by Jacobsen et al.^30^ On the contrary, the lower barriers recorded on TM/Mo(110) (TM = Pd, Rh, Ag, Pt, and Au), following Jacobsen’s principle, are rationalized by the weak adsorption energies of atomic N on the pure TM surfaces and strong binding on bare Mo(110) substrate. It can be noted that all TM-decorated Mo(110) alloys are less negative (and therefore less spontaneous) in reaction energy (−1.66 to −2.50 eV) than what has been found on bare Mo(110), signifying that the reaction is thermodynamically more favorable on the latter. Overall, the introduction of Ag, Au, Co, Cu, Fe, Ni, Pd, Pt, Rh, and Ru to the Mo(110) surface has helped significantly decrease the adsorption energies as shown in Table 2; nonetheless, only Ag, Au, Co, Pd, Pt, and Rh have resulted in lower activation barriers, with Pd exhibiting a maximum decrease by 0.21 eV.

#### 3.3.2 N_2_ Dissociation on TM/Fe(110) Alloys

**Figure 6:**
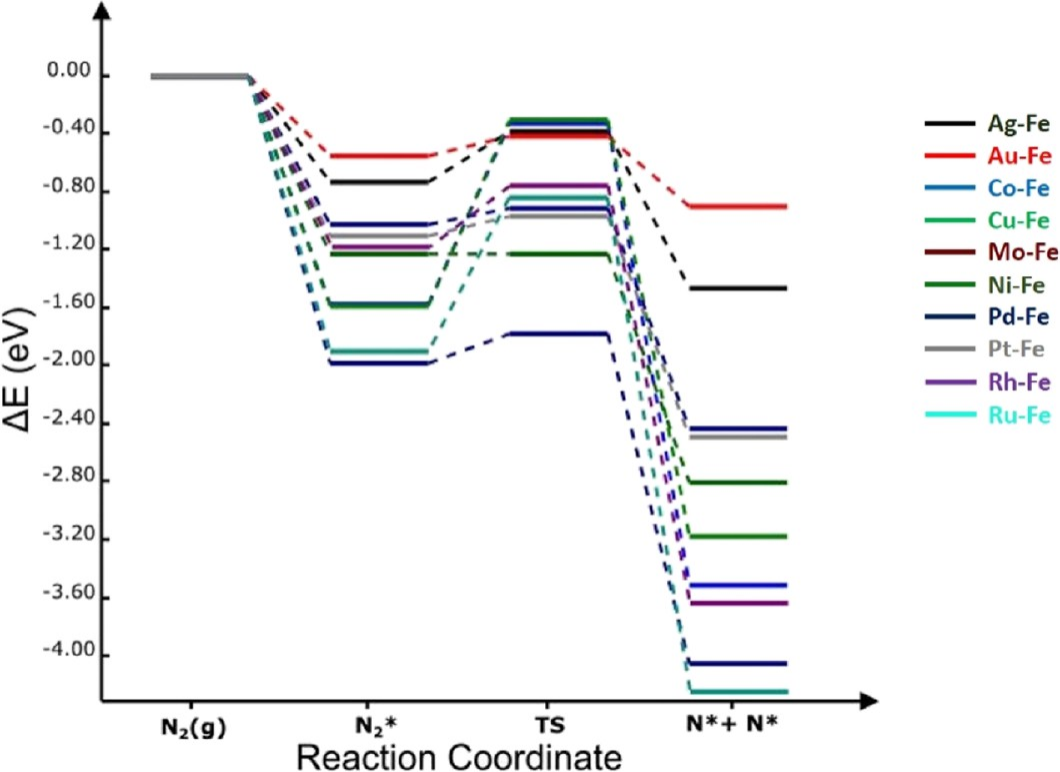
Potential energy of N_2_ dissociation process on **Fe-based alloy** surfaces at 0 K. The energies are computed with respect to the N_2(g)_ molecule.

The activity of Fe(110) catalytic surface could be also manipulated by inducing ligand effects so as to modify its surface electronic characteristics.^31^ Alloying the Fe(110) surface with other TM atoms is beneficial for constructing a tunable d-orbital electron that alleviates the sluggish kinetics for N_2_ activation. The IS, TS, and FS structures for the reaction pathway for N–N bond cleavage on all iron based alloy surfaces are displayed in Figures S7 and S8. Considering the TS structure on Fe-based alloy surfaces, the N–N bond was ruptured since the *d*_(N–N)_ length stretched from 1.17 Å on Ag/Fe(110), to 1.79 Å on Cu/Fe(110), with reference to the gas phase molecule. According to Figure 6, unlike TM/Fe(110) (TM = Co, Cu, Rh, and Ru), TM/Fe(110) (TM = Ag, Au, Mo, Pd, and Pt) surfaces exhibited a lower dissociation barrier with reference to the bare Fe(110) counterpart, signifying an excellent N_2_ activation activity. Interestingly, the least energy barriers obtained on Pd/Fe(110) (0.11 eV), Pt/Fe(110) (0.13 eV), and Au/Fe(110) (0.13 eV) surfaces following the Jacobsen’s principle^30^ because of the weak binding energies of atomic N on pure TM surfaces and the strong binding on Fe(110) surface. Apart from Mo, it can be noticed that the favorability of N_2_ dissociation, producing two atomic N, increases while moving from the Fe(110) substrate alloying with early to late transition metals. It should be noted that no transition state was found for the Ni/Fe(110) alloy surface, which implies that the N–N decoupling process is barrier-less. The origin of difference in activity of the considered Fe-based alloys can be attributed to the electronegativity difference between Fe and TM, which in turn plays a pivotal role in fluctuating d-states belonging to the surface atoms.^31^ The dissociation process is exothermic on all TM/Fe(110) surfaces, with dissociation energy (Δ*E*) values of Ru (−3.01 eV), Rh (−2.47 eV), Mo (−2.08 eV), Co (−1.93 eV), Cu (−1.59 eV), Ni (−1.57 eV), Pt (−1.43 eV), Pd (−1.41 eV), Ag (−0.73 eV), and Au (−0.35 eV). From a thermodynamic perspective, the process of N–N bond scission would take place more quickly on TM/Fe(110) (TM = Rh and Ru) than on the parent Fe(110) surface. All in all, the introduction of Ag, Cu, Co, Ni, Pd, Pt, and Rh helps weaken the interaction of the N_2_ molecule with the alloyed Fe(110) surface, with Au showing the lowest *E*_ads_ value of −0.55 eV. Nonetheless, the N_2_ dissociation barrier is optimized only for Ag, Au, Mo, Ni, Pd, and Pt, as shown in Figure 6.

#### 3.3.3 N_2_ Dissociation on TM/st.Ru(0001) Alloys

**Figure 7:**
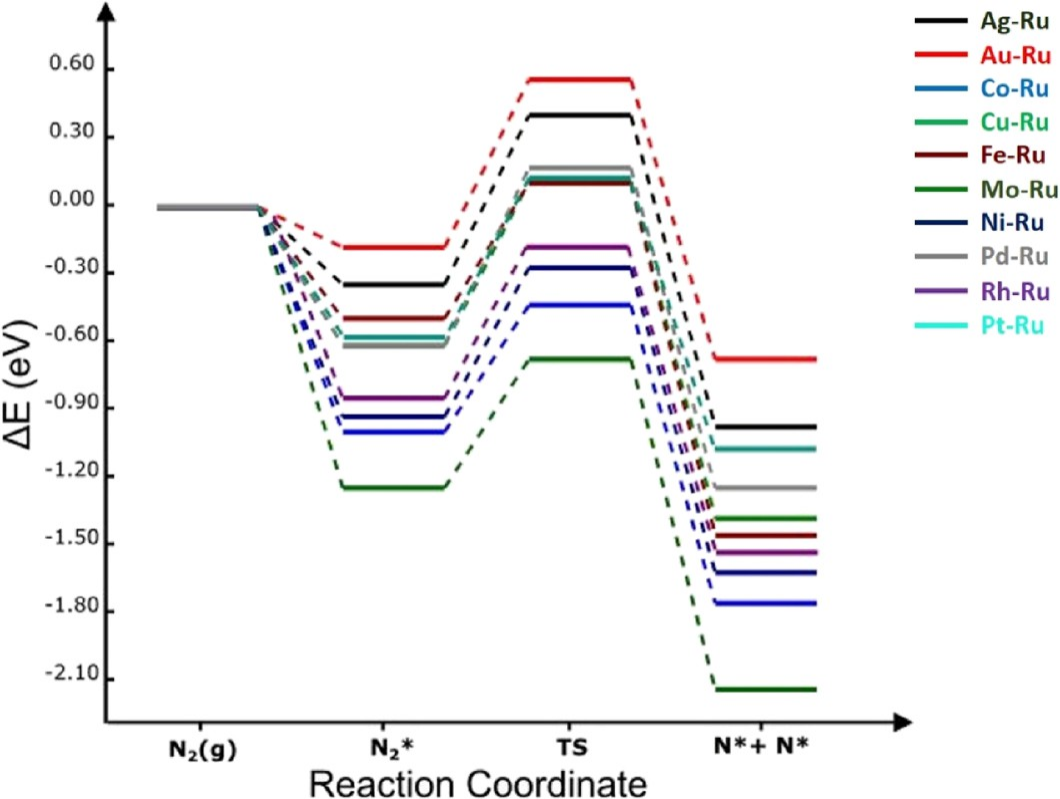
Potential energy landscape of N_2_ dissociation process on stepped **Ru-based alloy** surfaces at 0 K. The energies are computed with respect to the N_2(g)_ molecule.

The dissociation of N_2_ was also examined on TM/st.Ru(0001) alloys in order to scrutinize the influence of decoration of Ru steps with the TM promoter on the kinetic barrier for N–N decoupling. Figures S9 and S10 depict the dissociation pathway, i.e., IS, TS, and FS structures for all st.Ru-decorated surfaces, and the corresponding potential energy landscape is shown in Figure 7. Notably, the dissociation of N_2_ followed a similar reaction pathway on all step-decorated surfaces, wherein the N_2_ molecule sitting on the fivefold site, particularly at the bridge site between the step and terrace atoms, is directly dissociated in the TS as confirmed by the elongation of the N–N bond in the range of 1.79–1.85 Å on all TM covered-Ru surfaces. This five-atom feature was reported to offer a large degree of stabilization to the transition state (TS), thereby lowering the kinetic barrier.^32^ Subsequently, one N moves to the hybrid hollow hcp site of the step while the second N adsorbs at the hcp hollow site of the terrace in the FS structure. A similar pathway for N_2_ bond scission was reported in previous theoretical studies.^32^ The barrier for N–N bond cleavage followed the ascending order on TM/st.Ru(0001): Co (0.56 eV) < Mo (0.57 eV) < Fe (0.60 eV) < Ni (0.66 eV) < Pt = Rh (0.71 eV) < Cu (0.73 eV) < Au (0.74 eV) < Ag (0.75 eV) < Pd (0.79 eV). It is clear that only TM/st.Ru(0001) (TM = Co and Mo) alloy surfaces presented slightly lower dissociation barriers with reference to the bare st.Ru(0001). Mortensen et al.^33^ studied promoting Ru(0001) with Na and Cs using DFT computations and found that these alkali metals has only reduced the kinetic barrier, for N_2_ decoupling on bare Ru(0001), by 0.31 eV or less. The dissociation process is exothermic and spontaneous on all TM/st.Ru(0001) surfaces, with dissociation energy (Δ*E*) values of Fe (−0.96 eV), Mo (−0.89 eV), Co (−0.75 eV), Ni (−0.68 eV), Rh (−0.65 eV), Ag (−0.63 eV), Pd (−0.63 eV), Cu (−0.57 eV), Au (−0.50 eV), and Pt (−0.45 eV). Apparently, the dissociation process is thermodynamically more favorable on these bimetallic surfaces than that on the parent st.Ru(0001) which releases a lower reaction energy of −0.27 eV. It is speculated that the promotion of st.Ru(0001) with TM atoms has appreciably affected the thermodynamics of the dissociation process rather than the reaction kinetics. To briefly conclude, alloying the stepped Ru surface with all metals except Mo weakened the N_2_ interaction with the doped surface in comparison to the clean stepped Ru surface. Especially for Ag/st.Ru(0001) and Au/st.Ru(0001), the adsorption energies drastically decreased to −0.35 and −0.18 eV, respectively. Nonetheless, across all metals, the activation barrier was enhanced only in Mo/st.Ru(0001) and Co/st.Ru(0001).

### 3.4 Electronic Structure Analysis

#### 3.4.1 Charge Transfer Analysis

**Figure 8:**
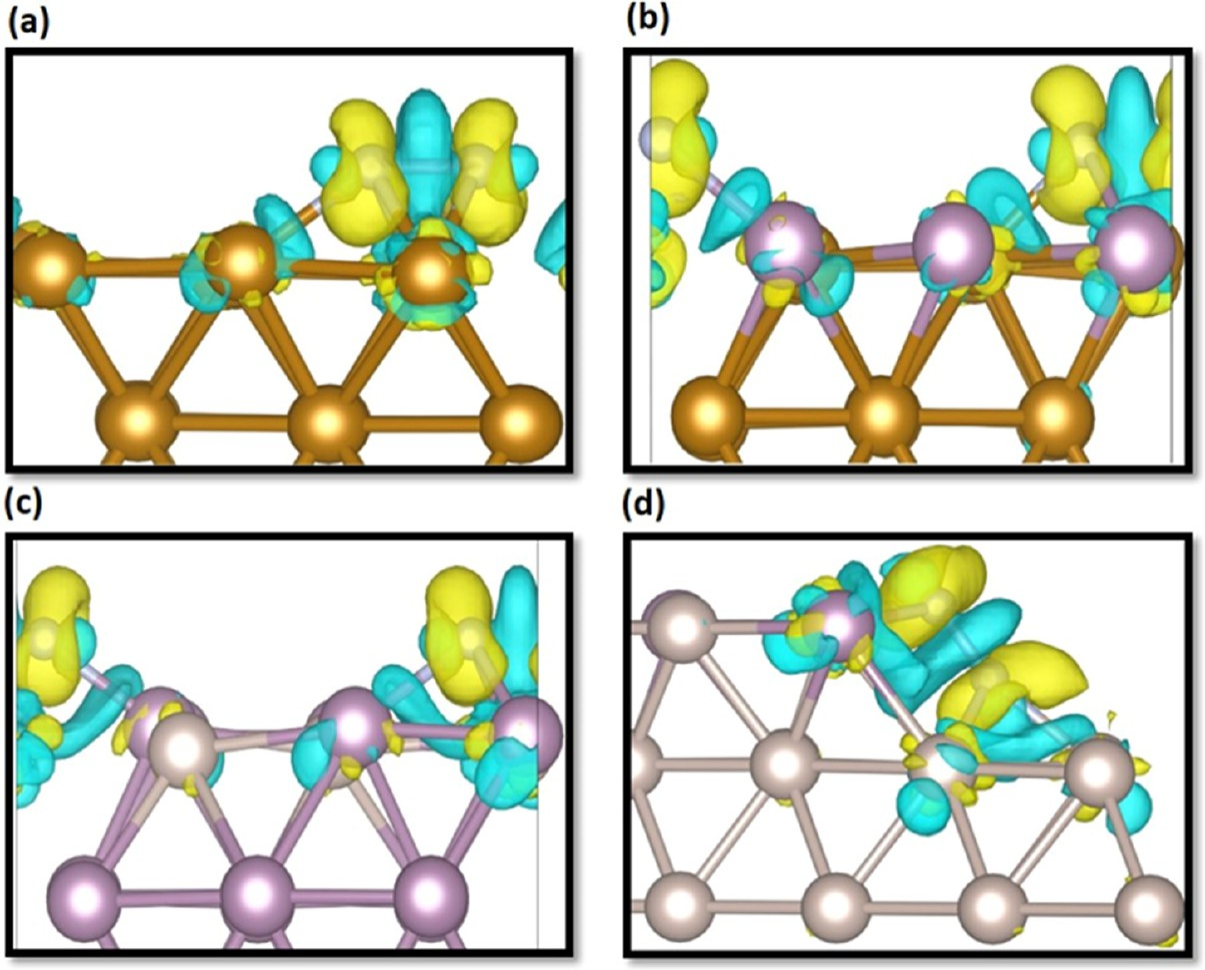
Charge density difference for most stable N_2_ adsorption configurations on (a) monometallic Fe(110), (b) Mo/Fe(110), (c) Ru/Mo(110), and (d) Mo/st.Ru(0001) surface. The iso-surface value is 0.005 e/bohr^3^. Yellow and blue colors correspond to charge accumulation and depletion, respectively.

The charge transfer from/to active sites could dramatically affect the binding energies and therefore alter the activity of the catalyst. This is directly related to the proportion of charged sites, which evolves as a consequence of the interaction with the guest molecule. In an attempt to clarify the adsorption behavior between N_2_ molecule and the surfaces under study, the charge density difference was obtained for the most stable conformations of N_2_ on various surfaces, as depicted in Figure 8. Clearly, the charge accumulation mainly appears around the nitrogen atoms, whereas charge depletion is found around the TM–N and N–N bonds. This charge transfer weakens the N≡N bond, thereby enhancing the decoupling of the N_2_ molecule. The N–TM bonds are formed via transfer of lone pair of electrons from d orbitals of the metal to N_2_ molecule π* antibonding orbitals. This electron acceptance/donation process is responsible for nitrogen activation.^20^ A Bader charge analysis was performed to quantify the charge transfer between the N_2_ molecule and various pure and alloyed surfaces. The direction of electron transfer is from the considered surfaces to the N_2_ adsorbate. Sizeable charge transfer was found for the most stable configurations of all alloyed surfaces, as tabulated in Table 3.

**Table 3:** Net Charge (*Q*) of N_2_ Molecule on Various TM/**Mo**(110), TM/**Fe**(110), and TM/**st.Ru**(0001) Alloyed Surfaces

solute TM metal	*Q*		
	TM/Mo(110)	TM/Fe(110)	TM/st.Ru(0001)
Ag	–0.99	–0.32	–1.68
Au	–1.39	–0.96	–1.85
Co	–0.95	–1.11	–1.06
Cu	–1.17	–1.07	–1.42
Fe	–2.14	-	–1.25
Mo	-	–1.22	–1.38
Ni	–1.36	–1.17	–1.06
Pd	–1.19	–0.31	–1.63
Pt	–1.37	–1.06	–1.09
Rh	–1.71	–1.13	–1.35
Ru	–1.26	–1.05	-

The charge transfer (|*Q*|) followed the descending order: Fe/Mo(110) > Rh/Mo(110) > Au/Mo(110) > Pt/Mo(110) > Ni/Mo(110) > Ru/Mo(110) > Cu/Mo(110) > Ag/Mo(110) > Pd/Mo(110) > Au/Mo(110), pointing out that the *Fe-functionalized Mo(110) surface presents the highest charge transfer of −2.14 |e| to N*_2_*molecule*. Notably, the N_2_ molecule acquires more electrons from the Mo/Fe(110) surface as compared to other Fe-based alloy surfaces, surmounting for −1.22 |e|. This high charge transfer gives rise to the highest elongation (18%) of N–N bond length of the N_2_ molecule on the Mo/Fe(110) surface, as shown in Table 2, thereby promoting the N_2_ activation step. On the other hand, the little charge transfer from Ag/Fe(110) and Pd/Fe(110) surfaces has resulted in stretching the N–N bond length by only 1.8%. It can be inferred that the elongation of the N–N bond distance of N_2_ adsorbate was caused by charge transfer from TM d states to antibonding orbitals of N_2_.^34^ A similar finding was reported by Song et al.^20^ for N_2_ adsorption over TM-doped Ir(100) surfaces.

Considering the cases of **st.Ru-based** alloys, Au- and Ag-decorated substrates facilitated higher charge transfers of −1.85 and −1.68 |e|, respectively, toward N_2_ compared to other considered surfaces. The increased negative charge and N–N bond length implies reductive activation of N_2_ adsorbate through an improved π back-donation from the metal centers, as depicted in the charge density difference shown in Figure 8.

It is clear that doping the monometallic surface with another TM element (heterometal) results in dispersion of the host surface’s atoms, thereby leading to reduction in number of host TM sites (ensemble effect). The Bader charges of the interacting atoms of the N_2_ adsorbate and various surfaces for the adsorption structures (Figure 8) are presented in Table 4. The charge of the two N atoms (N1 and N2) appears more electronegative on the studied catalytic surfaces as compared to the N_2_ gas-phase state. This further supports the finding of electron transfer from the surfaces to the adsorbate. Notably, the interacting Fe atoms possess 0.08 and 0.14 e on the Fe(110) surface; however, on the Mo/Fe(110) surface, these Fe atoms become more negatively charged, which implies that N_2_ withdraws electrons mainly from Fe atoms.

**Table 4:** Bader Charges of the Interacting Atoms (N1 and N2, Given in Parentheses) of the N_2_ Molecule and the Metallic Surface

surface	Bader charge	
	adsorbate	surface
gas phase	0.17 (N1)	-
	0.13 (N2)	-
Fe(110)	–0.62 (N1)	0.14 (Fe)
	–0.83 (N2)	0.08 (Fe)
Mo/Fe(110)	–0.59 (N1)	0.33 (Mo), −1.96 (Fe)
	–0.63 (N2)	1.04 (Mo), −2.07 (Fe)
Ru/Mo(110)	–0.63 (N1)	3.90 (Mo)
	–0.63 (N2)	3.24 (Mo)
Mo/st.Ru(0001)	–0.86 (N1)	3.24 (Ru), 3.47 (Mo)
	–0.52 (N2)	3.15 (Ru)

#### 3.4.2 Density of States

**Figure 9:**
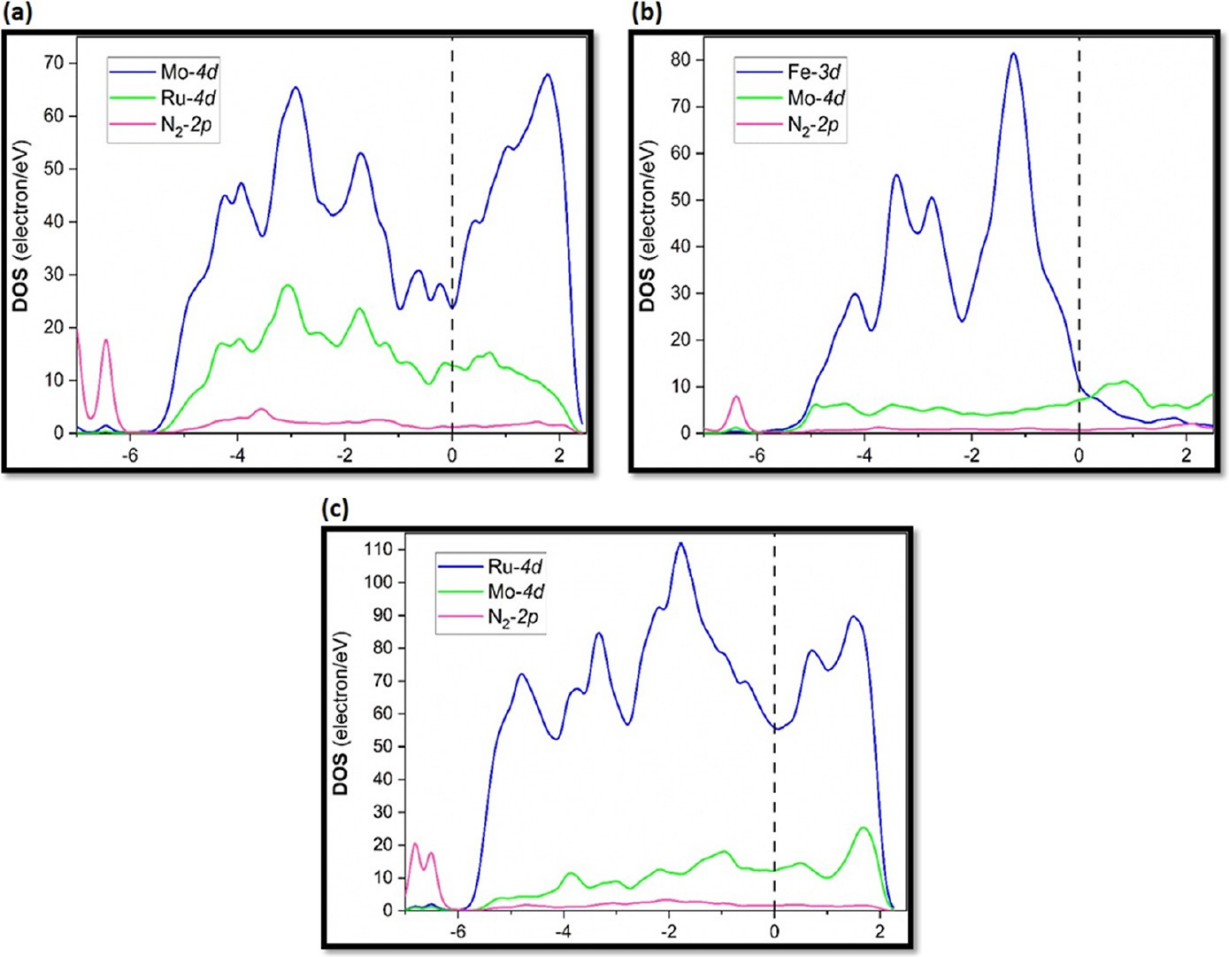
PDOS of N 2p and TM 4d for N_2_ binding on (a) Ru/Mo(110), (b) Mo/Fe(110), and (c) Mo/st.Ru(0001). The dashed vertical line represents the Fermi level (*E*_F_).

Although the Bader charge analysis revealed that electrons transferred between the different alloy surfaces and N_2_ molecules, the distribution of these transferred electrons in the molecule and the surface is still unclear. The transferred electrons play a key role in the N≡N bond activation; thus, it is essential to study their distribution. Therefore, PDOS between N and constituent metal atoms (in pure and alloyed surfaces) were analyzed for the *energetically most favorable N*_2_*adsorption configurations* on the three types of alloy systems (considering only the most stable ones), namely, Ru/Mo(110), Mo/Fe(110), and Mo/st.Ru(0001), as displayed in Figure 9. It is clear that N_2_ adsorbate possessed an electronic state occupied by 2p orbital close to the Fermi level (*E*_F_) of −6.5 eV, which agrees well with the value of −7.5 eV reported by Song et al. for N_2_ binding on TM-doped Ir(100) surfaces.^20^ This implied that the originally empty π antibonding orbital in nitrogen molecule was activated upon electron transfer. Clearly, there are two small hybridization peaks between N 2p and Mo 4d as well as N 2p and Ru 4d at nearly −6.4 and −7.0 eV, while no hybridization was recorded between N 2p and Fe 3d. These findings are consistent with the charge transfer results (Table 3), which revealed higher charge transfer between the N_2_ molecule and Ru/Mo(110) and Mo/st.Ru(0001) as compared to the Mo/Fe(110) substrate. It is noteworthy that the higher d-state density recorded on Mo/st.Ru(0001) at the Fermi energy level (*E*_F_) facilitated a charge transfer of −1.38 toward N_2_ as compared the other considered alloy surfaces (Figure 9), such as Ru/Mo(110) (−1.26) and Mo/Fe(110) (−1.22).^35^

**Figure 10:**
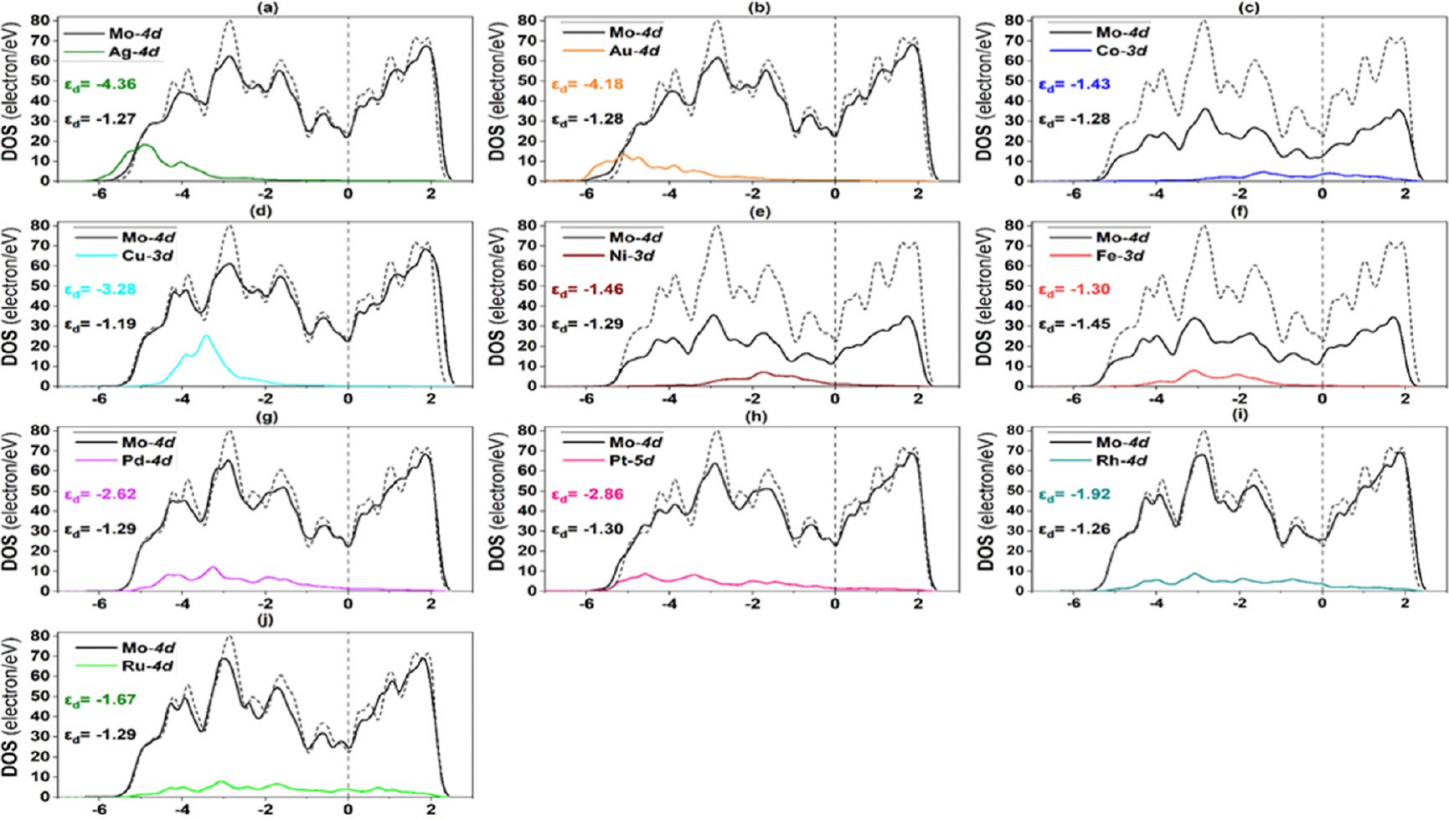
PDOS of **Mo(110) alloy** surface constituting TM: (a) Ag, (b) Au, (c) Co, (d) Cu, (e) Ni, (f) Fe, (g) Pd, (h) Pt, (i) Rh, and (j) Ru. The dashed and short lines dashed represent the Fermi level (*E*_F_) and monometallic Mo(110) surface, respectively.

**Figure 11:**
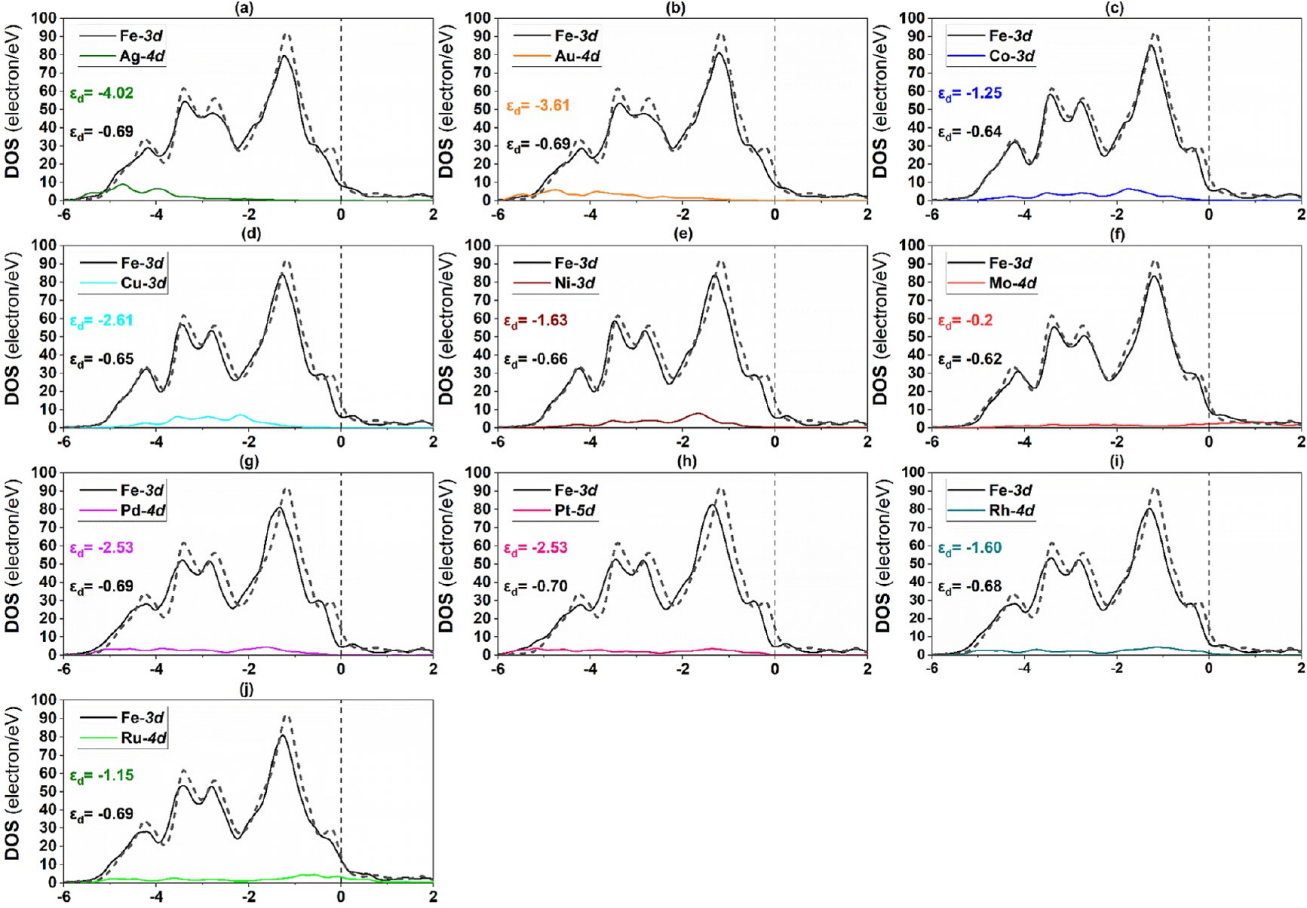
PDOS of Fe(110) alloy surface constituting TM: (a) Ag, (b) Au, (c) Co, (d) Cu, (e) Ni, (f) Mo, (g) Pd, (h) Pt, (i) Rh, and (j) Ru. The dashed and short lines dashed represent the Fermi level (*E*_F_) and monometallic Fe(110) surface, respectively.

**Figure 12:**
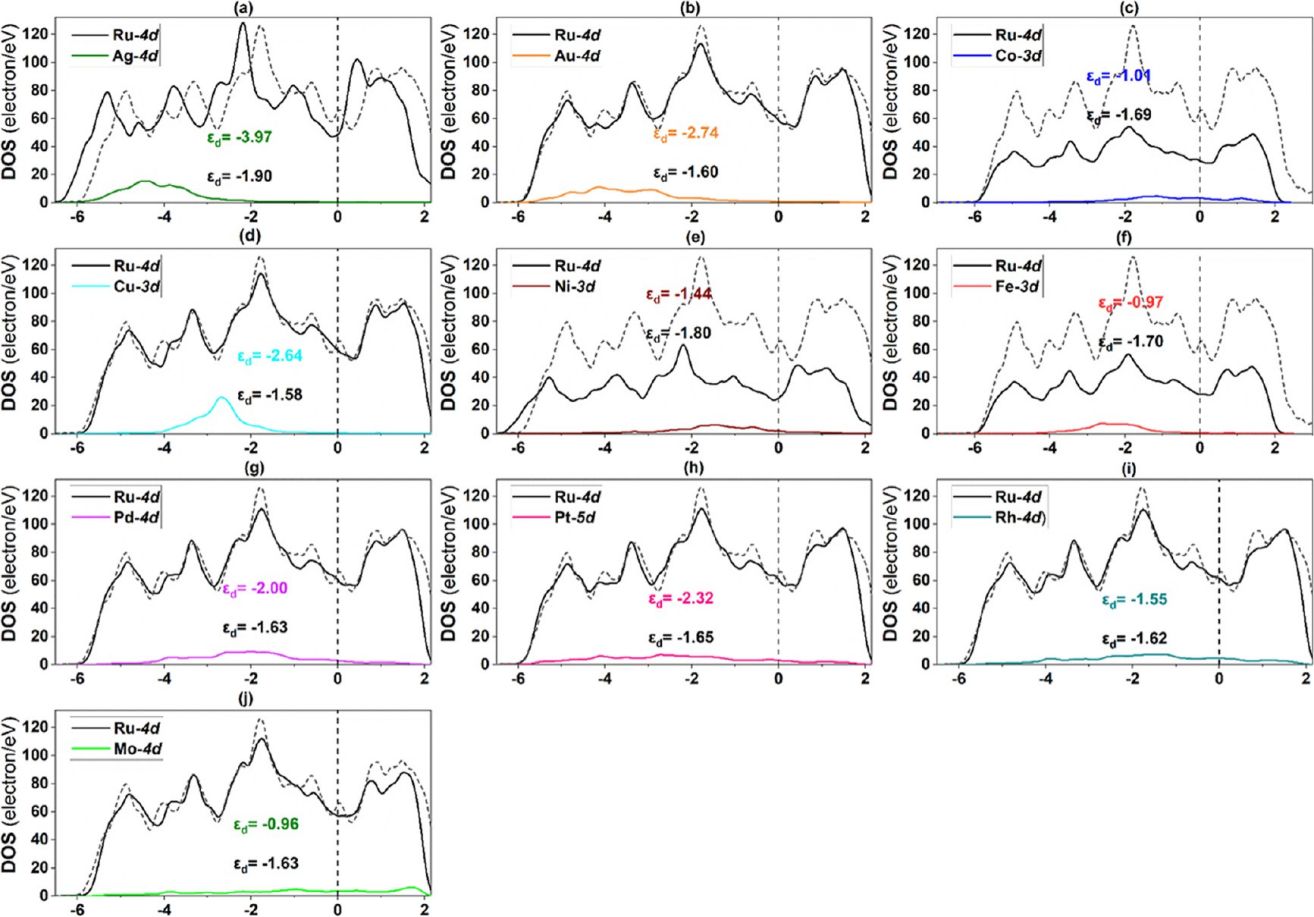
PDOS of stepped Ru(0001) alloy surface constituting TM: (a) Ag, (b) Au, (c) Co, (d) Cu, (e) Ni, (f) Fe, (g) Pd, (h) Pt, (i) Rh, and (j) Mo. The dashed and short dashed lines represent the Fermi level (*E*_F_) and monometallic stepped Ru(0001) surface.

The PDOS of Mo 4d, Fe 3d, Ru 4d, and TM 4d (or 3d) were also explored in order to clarify the electronic structure of TM-decorated alloy surfaces. The PDOS of the TM/Mo(110) alloy surfaces and the monometallic Mo(110) surface are shown in Figure 10. A more effective hybridization of Mo 4d and TM 4d (or 3d) was noticed in the energy range of −6 to 0 eV depending on the type TM metal, indicating the synergistic effect between TM and Mo atoms. Song et al. revealed a similar observation for TM-doped Ir(100) surfaces, wherein hybridization of TM 3d and Ir 5d orbitals was found in the range of −5 to −2.5 eV.^20^ This hybridization gives rise to charge transfer, which is essential for activating the N_2_ molecule, as discussed above. It is evident that the hybridization is more pronounced in TM/Mo(110) alloy surfaces as compared to Fe- and st.Ru-based surfaces, as displayed in Figures 11 and 12, respectively. According to Figure 11, the d orbitals of Fe and Au on the Au/Fe(110) surface have a hybridization interaction between −5 and −2 eV, which coincides well with the literature values.^36^ It is clear that the d-orbital peaks of the Mo(110), Fe(110), and st.Ru(0001) (short dashed lines) monometallic surfaces cross the Fermi level, which implies that they could readily donate electrons for nitrogen adsorption.

**Figure 13:**
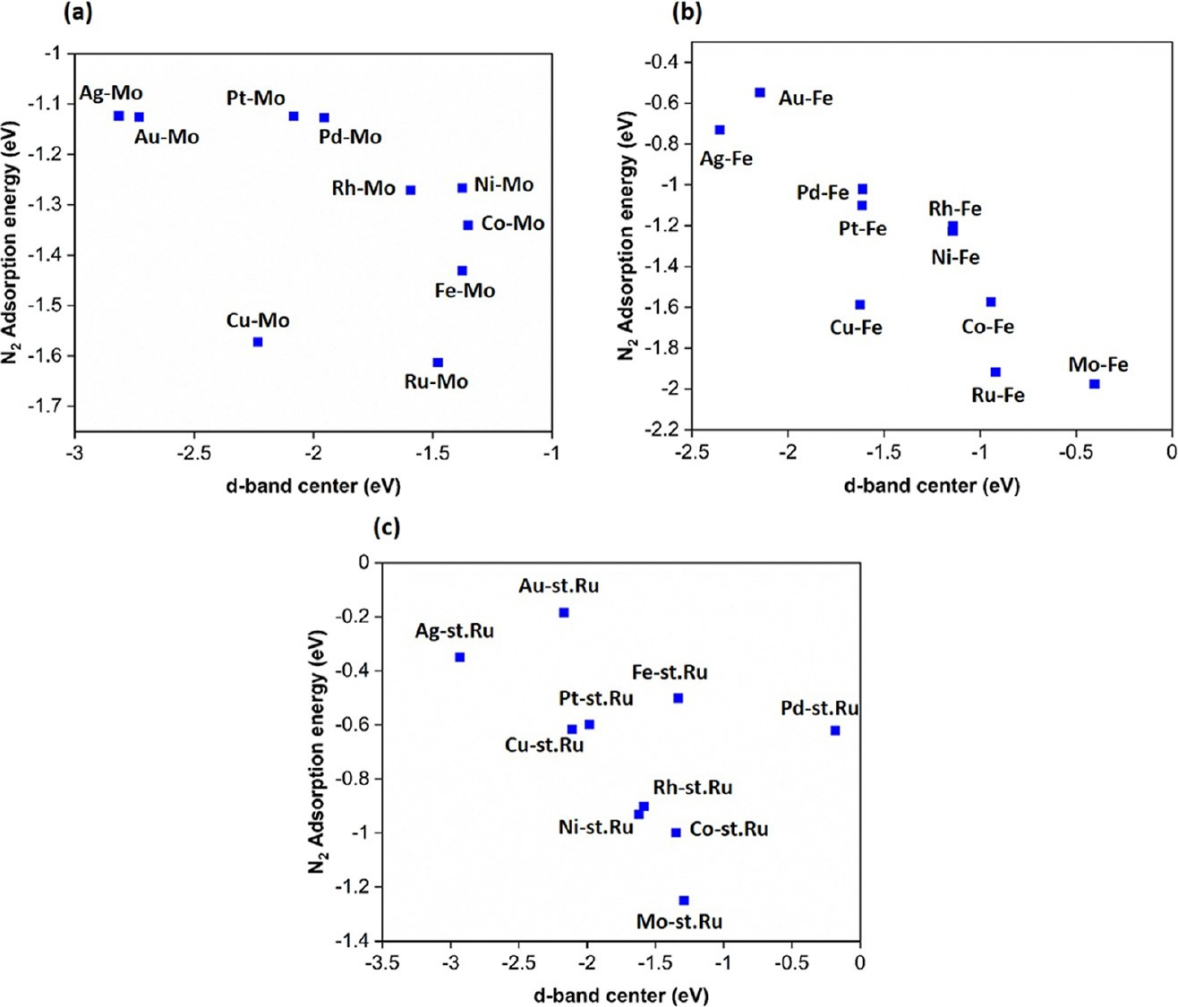
N_2_ binding energy plotted against the position of d-band center of (a) Mo(110), (b) Fe(110), and (c) st.Ru(0001) alloy surfaces constituting TM dopants.

To unveil the reason behind the obtained adsorption strength trends of the N_2_ molecule on TM-based Mo(110), Fe(110), and TM/st.Ru(0001) surfaces, the position of the d-band center was calculated for both host and TM solute atoms on all bimetallic surfaces (Figures 10–12), and the average d-band center for all bimetallic surfaces is plotted against N_2_ binding energy (Figure 13). Notably, the d-band centers of Ag, Au, Pd, and Pt, on the three alloys types, are more downshifted from the Fermi level in comparison to other TM solute atoms. On the other hand, the d-band center values for Fe, Mo, Ru, Ni, and Co solute atoms are shifted close to the Fermi energy level; therefore, strong binding energies of N_2_ are observed on all bimetallic surfaces containing these TM atoms. This can be explained by the d-band theory, wherein the higher d-band center of a metallic substrate results in a fewer occupied antibonding orbitals when the p-orbitals of an adsorbate approach the surface, therefore resulting in a stronger adsorption with the adsorbate.^36^ Overall, it can be inferred that the d-band center moved toward the negative direction for all late-TM based alloys, thereby allowing the nitrogen molecule to adsorb weakly as compared to the early-TM surface alloys. A similar trend was observed by Das et al.^31^ for N adsorption on Fe(110) surface alloyed with TM (=V, Ti, Sc, Cu, Mn, Ni, Co, and Cr).

### 3.5 N_2_ Adsorption under Mechanical Strain Effect

**Figure 14:**
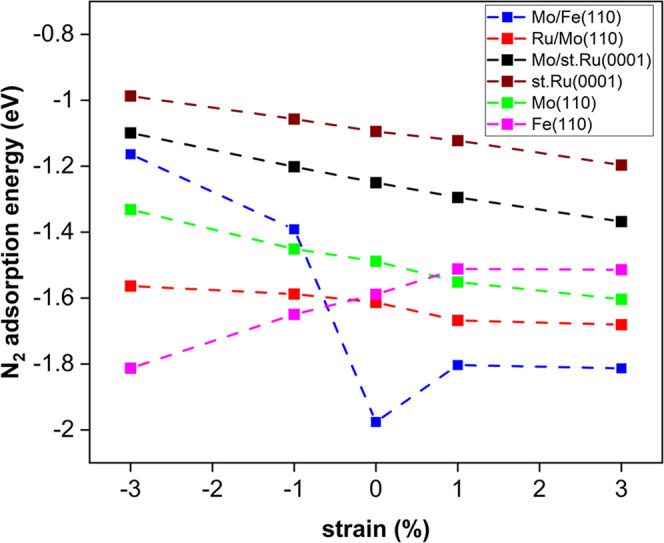
N_2_ adsorption energy (eV) on Ru/Mo(110), Mo/Fe(110), Mo/st.Ru(0001), Mo(110), Fe(110), and st.Ru(0001) surfaces under strain effect.

Strained catalytic surfaces can be found in multielemental metallic catalysts owing to the lattice mismatch between the constituent metals. Such strains could significantly improve the chemisorption characteristics of the catalyst, either for intermediates produced during the course of reaction or for the adsorbate.^37^ This is because the strain modifies the surface ability to form bonds; thus, strain could be regarded as catalyst’s reactivity modifier. For example, the introduction of mechanical strain through ball milling of the catalyst has been experimentally proven to affect the catalyst activity and behavior.^12^ In this context, the influence of compressive (−3 and −1%) and tensile strains (3 and 1%) on binding strength of N_2_ molecule was examined for **three types of alloy systems**, having strong N_2_ adsorption energy, namely, Ru/Mo(110), Mo/Fe(110), and Mo/st.Ru(0001) surfaces, and their monometallic counterparts. The chosen adsorption site was exactly the same as that on the corresponding unstrained surface. Figure 14 depicts that Ru/Mo(110), Mo/Fe(110), and Mo/st.Ru(0001), along with the monometallic Mo(110) and st.Ru(0001) surfaces followed the same pattern; as the biaxial strain increases and moves from compressive to tensile, the N_2_ binding energy becomes stronger. For most of the alloy surfaces studied, apart from the Mo/Fe(110) one, the N_2_ binding energy on the previously mentioned surfaces varies as a linear function of the applied strain. Kattel and Wang^38^ studied the adsorption of oxygen reduction reaction (ORR) species on strained Pt(111) surface, and the results revealed that ORR species adsorbed more weakly on the compressively strained substrates in comparison to the unstrained surface. This behavior was ascribed to downshifting of the d-band center caused by the decrease in the interatomic separation distances between Pt atoms. On the contrary, strained Fe(110) presented stronger adsorption energies of N_2_ molecule under compressive strain (*E*_ads_ = −1.81 to −1.65 eV) as compared to tensile strain (*E*_ads_ = −1.51 eV). It can be speculated that if the change in the lattice parameters is relatively large, considerable tuning of the adsorption at the same adsorption site is expected.

At the same time, it is known that heterometallic bonding interactions, termed “chemical strain effect”, between the solute and host atoms could lead to modification of the lattice constants in bimetallic alloy surfaces.^39^ However, it is difficult to decouple chemical and mechanical strain effects because they take place together. The influence of chemical strain, in the absence of mechanical strain, on lattice constants (**a and b**) was explored for the three alloy types as tabulated in Tables S8–S10. Apparently, functionalizing the Mo(110) and st.Ru(0001) surfaces with Co and Cu atoms resulted in significant contraction of both **lattice constant a** (from −6.50 to −2.18%) and **b** (from −6.70 to −3.56%). Conversely, alloying Mo(110) and Fe(110) alloy systems with Pd and Pt has led to considerable elongation in **a** and **b lattice parameters** from 0.38 to 0.98% and from 0.53 to 0.98%, respectively.

### 3.6 Establishing Periodic Trends

#### 3.6.1 Brønsted–Evans–Polanyi

**Figure 15:**
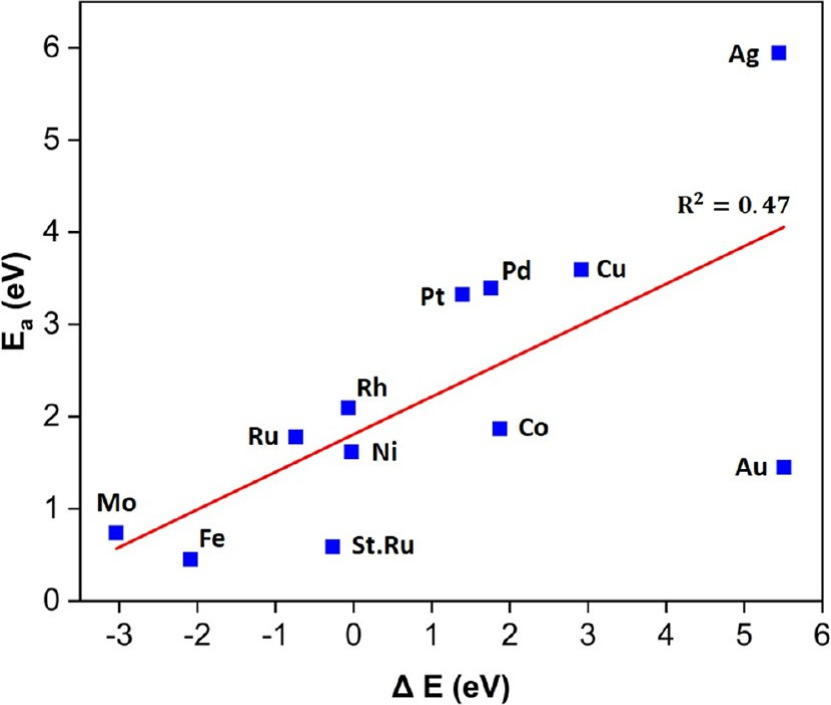
BEP relationship for N_2_ dissociation on various monometallic surfaces.

It is well established that there is a linear relationship between activation barrier and reaction energy of surface reactions over TM substrates, which is called Brønsted–Evans–Polanyi (BEP) relation.^40,41^ This relationship has been commonly adopted in heterogeneous catalysis because it can significantly reduce the time and efforts to determine the activation barrier. Herein, the BEP relation was obtained for N_2_ dissociation by investigating the relation between the activation barrier and reaction energy ***over 12 monometallic surfaces***, as depicted in Figure 15. The linear BEP relation can be written as follows: *E*_a_ = 0.41Δ*E* + 1.81. This implies that the kinetic barrier of N_2_ dissociation can be easily determined from the reaction energy. It is clear that the activation energy values on Mo(110), Rh(111), Ni(111), and Ru(0001) fit well into the scaling relationship.

#### 3.6.2 Closer Look into the Fe-, Mo-, and Ru-Based Alloys

##### 3.6.2.1 Fe-Based Alloys

Interestingly, it can be noticed that alloying the bare Fe(110) substrate with **early TM** (=Co, Cu, Mo, Rh, and Ru) atoms presents stronger binding energies of atomic N in comparison to that of the bare Fe(110) substrate, thereby inhibiting the diffusion away from the catalytic site. This could lead to quick deactivation of the surface and therefore sluggish adsorption process.^42^ Conversely, Fe alloying with **late-TM** (=Ag, Pd, Pt, and Au) shows a wide range of binding energies with an overall tendency to weaken its N adsorption with reference to the monometallic Fe(110) surface. Therefore, it can be inferred that TM-decorated Fe(110) alloys with early TM, except for Ni, can be placed in the strong adsorption regime for the adsorbed N atom, whereas the late-TM alloys presented a relatively weaker binding tendency. A similar finding was reported by Das et al.^31^ for atomic N adsorption on Fe(110) surface alloyed with TM (=Sc, Ti, V, Cr, Mn, Co, Ni, and Cu). They have found that doping the Fe(110) surface with early TM (Sc to Mn) has resulted in higher adsorption energies for atomic N with reference to that of the bare substrate. On the contrary, Fe alloys substituted with late transition metals (Co to Cu) showed weaker binding in comparison with their monometallic Fe(110) counterpart. The observed binding strength was ascribed to the d-band center values for late-TM-decorated alloys experiencing a downshift toward the negative direction, enabling the atomic N to adsorb weakly in comparison to the bare Fe(110) and early-TM substituted alloys as well.

It is noteworthy that the N_2_ dissociation barrier decreases considerably on TM/Fe(110) alloy surfaces while moving from early to late TM, with the exception of Mo dopant. An opposite trend was reported by Pozzo and Alfè^42^ for H_2_ dissociation on TM-doped Mg(0001), and they have found that promoting the Mg(0001) substrate with TM on the left of the periodic table eliminates the kinetic barrier for N_2_ decoupling; however, the effect of transition metals on the right is small. The have correlated these opposite trends to the position of the d-band center of the TM dopant.

##### 3.6.2.2 Ru-Based Alloys

In regard to the charge transfer analysis, apart from Pt, Cu, and Mo, the degree of charge transfer increased remarkably on TM/st.Ru(0001) alloy surfaces when going down the periodic table. In addition to the abundance of nonbonding electrons, this could be ascribed to the fact that ionization potential decreases upon going down through the periodic table, thereby the d metal electrons can be easily moved to the N_2_ adsorbate. It can be concluded that a wider range of dissociation barrier and adsorption energy values were obtained while doping the host metal surface with early and late TM, which signifies that the strength of interaction between adsorbate species and surface atoms is sensitive to chemical composition of the catalytic surface.

##### 3.6.2.3 Mo-Based Alloys

The process of breaking the N–N bond on Mo-based alloy surfaces follows two governing patterns depending on the heterometal; first, for TM/Mo(110) (TM = Pd, Rh, Ag, Pt, Au, Co, Ni, and Cu), the N_2_ molecule is positioned on the hollow site of the IS structure. The two nitrogen atoms of FS structure are situated on the hollow site. Second, for TM/Mo(110) (TM = Ru and Fe), the IS structure presents the dissociated nitrogen atoms of the FS structure to be adsorbed on the hybrid hollow site, formed by one TM (TM = Ru and Fe) atom and two Mo atoms. The dissociation barrier increased in the following order on TM/Mo(110) alloys: Pd (0.43 eV) < Rh (0.51 eV) < Ag (0.52 eV) < Pt (0.60 eV) < Au (0.61 eV) < Co (0.63 eV) < Ni (0.64 eV) < Cu (0.67 eV) < Ru (0.77 eV) < Fe (1.00 eV).

## 4 Conclusions

The high activation barrier required for cleavage of the N≡N bond during the Haber–Bosch process demands extreme reaction conditions. This issue could be circumvented by the functionalization of TM catalysts via the inclusion of different TM dopants. In this work, DFT computations are performed for Mo(110), Fe(110) and stepped Ru(0001) surfaces alloyed with wide range of TM heteroatoms (Ni, Co, Ru, Fe, Mo, Rh, Au, Ag, Pd, Pt, and Cu) to understand the N_2_ activation and dissociation steps; the monometallic surfaces are also considered as references. To begin with, the energetic stability of binary metal alloy surfaces was investigated using the formation energy as a major descriptor. Overall, TM-decorated Fe alloys exhibited relatively higher stability as compared to that of TM/Mo(110) and TM/st.Ru(0001) surfaces. Then, the adsorption of N_2_ molecule on bare metal and bimetallic (alloy) surfaces was carefully investigated with the analysis of adsorption energetics. The results revealed that the most favorable N_2_ adsorption conformations on the three types of alloy systems are Ru/Mo(110), Mo/Fe(110), and Mo/st.Ru(0001). The analyses of electronic characteristics (PDOS and charge transfer) revealed that high N_2_ activation on these bimetallic surfaces was mainly due to hybridization between the host and TM solute orbitals. This hybridization gave rise to considerable charge transfer from the surface to the antibonding orbital of nitrogen, which further weakened the triple bond and thereby promoted N_2_ decoupling. The dissociation barriers for N_2_ on monometallic and bimetallic substrates were also computed. It was found that all Mo-alloys, except for TM/Mo(110) (TM = Ru and Fe) surfaces, presented energy barriers lower than that of the bare Mo(110) surface. Interestingly, apart from Mo, the favorability of N_2_ dissociation increased while moving from the Fe(110) substrate alloying with the early to late TM series. The origin of difference in activity of the considered Fe-based alloys can be attributed to the electronegativity difference between Fe and TM, which in turn plays a pivotal role in fluctuating the d-band center belonging to the surface atoms. Notably, the promotion of st.Ru(0001) with TM atoms has appreciably affected the thermodynamics of dissociation process rather than the reaction kinetics. This work not only introduces efficient catalysts for nitrogen activation and decoupling but also provides valuable insights on the rational design of bimetallic catalysts to enhance the performance of the Haber–Bosch process for NH_3_ production.

## Supporting Information Available

N_2_ adsorption sites on monometallic and bimetallic surfaces; N_2_ and N adsorption on monometallic surfaces; Mo- and Ru-based alloys total energies; N_2_ adsorption energies on Fe- and Mo-based alloy surface sites; N_2_ dissociation on pure metals; chemical strain on Fe-, Mo-, and Ru-based alloys; N_2_ adsorption on Fe-based alloy; N_2_ adsorption on Ru-based alloy; N_2_ dissociation IS, TS, and FS on Fe-based alloys; and N_2_ dissociation IS, TS, and FS on st.Ru-based alloys (PDF)
