# Supplementary material for: Engaging the Concepts of Bimetallicity and Mechanical Strain for N2 Activation: A Computational Exploration
Source: ACS Appl Mater Interfaces. 2024 Oct 7;16(41):56254–70. doi: 10.1021/acsami.4c09691 (PMC11492178; doi:10.1021/acsami.4c09691)
Supplement: Supplementary file 1 — am4c09691_si_001.pdf [file am4c09691_si_001.pdf]

SUPPORTING INFORMATION

Engaging the concepts of bimetallicity and mechanical strain for N_2 activation: a computational exploration

Omer Elmutasim[1,2], Louai Mahdi Maghrabi[1,2], Dattatray S. Dhawale[3], Kyriaki Polychronopoulou[1,2*]

[1]Department of Mechanical and Nuclear Engineering, Khalifa University of Science and Technology, Main Campus, Abu Dhabi, P.O. Box 127788, UAE

[2]Center for Catalysis and Separations (CeCaS), Khalifa University of Science and Technology, Main Campus, Abu Dhabi, P.O. Box 127788, UAE

[3]CSIRO Energy, Private Bag 10, Victoria, Clayton South 3169, Australia

*Corresponding Email: Kyriaki.polychrono@ku.ac.ae

1. N_2 Adsorption on Monometallic surfaces

Geometry of Sites for adsorption: To determine the catalytic activity of the transition metals in the dissociation reaction step of N_2 molecule, DFT computations of the binding of N_x(x=2,1) species have been performed on the 12 monometallic surfaces in order to identify the most favorable adsorption site geometry. Four adsorption sites were chosen for bcc (110) structures; 3-fold hollow, short bridge, long bridge and top site (depicted in **Figure S1(a)** (**Supporting Information**)). Concerning the fcc(111) and hcp(0001) metals, four adsorption sites were considered; ***particularly fcc hollow, hcp hollow, 2-fold bridge and top site*** (shown in **Figure S1(b)**).

The binding energies and site preferences on monometallic surfaces are shown in Tables **S1 and S2, Supporting Information**). The 3-fold hollow site is mostly favored for both molecular and atomic nitrogen on pure metal surfaces owing to its high coordination number (CN).

The N_2 adsorption energies exhibit a monotonically decreasing trend as follows: Fe > Mo > Co > st.Ru > Ru > Rh > Cu > Pt > Pd > Au > Ni > Ag surfaces. Clearly, N_2 molecules prefer to be physisorbed on the most noble surfaces furthest to the right i.e. Ag(111) and Au(111), as depicted in Figure S2 (**Supporting Information**), whereas they are favorable to chemisorption on the rest of the surfaces. Tafreshi et al.[1] have also reported that N_2 interacts weakly with Cu(111) and the

molecule migrates away from the surface by ~ 3. Å. Lykke et al.[2] have reported that nitrogen adsorbs extremely weakly on Au(111) based on molecular beam-surface scattering experiments. Mark and co-workers[3] have also reported a similar physisorbed state of nitrogen on Au(111) with physisorption energy of -0.105 eV. The strength of physisorption is comparable to that of chemisorption. The physisorbed N_2 molecules are parallel to the previously mentioned surfaces with distances above 3 Å. In the most stable configurations of physisorbed N_2, the N-N bond distance ($d_{(N-N)}$) are 1.11 Å which are identical to the N_2 molecule in the gas phase, signifying a weak interaction between the N_2 and the surfaces under study. The weak interactions with these surfaces indicate that nitrogen could readily desorb by increasing the temperature. **Table S1** (**Supporting Information**) reveals that the N_2 molecule binds only weakly on Ag(111), Au(111) and Ni(111) surfaces with binding energy of -0.13, -0.27 and -0.15 eV, respectively. Notably, a relatively strong adsorption of N_2 molecule was recorded on Mo(110), Fe(110) and st.Ru(0001) surfaces, surmounting for adsorption energies of -1.49, -1.59 and -1.10 eV, respectively. Expectedly, the N_2 adsorption is more favorable on stepped Ru(0001) surface than its corresponding flat counterpart, wherein the N_2 binds at the step edge site (i.e. five-fold site), wherein the Ru atoms forming an irregular five-atom pentagon on the step, (-1.10 eV) on the stepped surface while Ru-top site (-0.78 eV) is the preferred site on the flat one. This can be ascribed by the lower coordination of the stepped surface atoms as compared to the terrace site ones.[1] Moreover, the high stability of N_2 binding on the stepped surface can also be ascribed to the unique 5–coordinated bonding of N_2 with five Ru atoms (five-fold site) from both the surface (step) and subsurface (terrace) layers as compared to one N-Ru bonding on the planar counterpart surface. The strong adsorption at uncoordinated step sites was noticed previously by Dahl et al.[4] who found that the binding of diatomic nitrogen at the step edge is more stable by 0.60 eV than that on terraces, an observation later proved experimentally by Morgan et al.[5]. This difference in reactivity between the terraces and steps implies that the rate of $N\equiv N$ bond cleavage is totally controlled by the little proportion of steps on the stepped Ru(0001) surface. Accordingly, the reaction of ammonia synthesis is extremely structure sensitive on ruthenium catalyst, since N_2 bond cleavage is the rate limiting step. Notably, the difference binding energies for atomic N on planar (-0.898 eV) and stepped Ru(0001) (-0.899 eV) surfaces are quite small (listed in Table S3 (**Supporting Information**)), which agrees well with results reported by Dahl et al.[4] for N binding on Ru(0001) surface. This finding also coincides well with the scanning tunneling microscopy (STM) observation that atomic N does not block the step sites on Ru(0001) after the adsorption of NO at room temperature.[6] Moreover, the hcp threefold-hollow site is the most preferable site for atomic N adsorption on Ru(0001) (Table S3, **Supporting Information**)), which

aligns with the experimental STM results, reported by Jacobi et al.[7], that N prefers to occupy hcp sites on Ru(0001) surface.

Functionalization of TM parent surface, via inclusion of TM hetero-atoms, could be the key to improve N_2 adsorption strength, which in turn might open possible low energy pathways for N_2 decomposition reaction. Since Mo(110), Fe(110) and stepped Ru(0001) surfaces presented a strong N_2 chemisorption behavior among the studied monometallic TM surfaces, further investigation of N_2 adsorption and dissociation was carried out for the bimetallic TM/Mo(110), TM/Fe(110) and stepped TM/st.Ru(0001) alloys by introducing the TM hetero-atoms into the outmost layer of the pure Mo(110), Fe(110) and st.Ru(0001) substrates (host structures). The host of these bimetallic structures are Mo-, Fe-, and Ru- coinciding quite well with the N_2 fixation in nature and the industrial practices, which are catalysed by nitrogenases (Fe-Mo enzymatic site) and Ru catalysts, respectively.

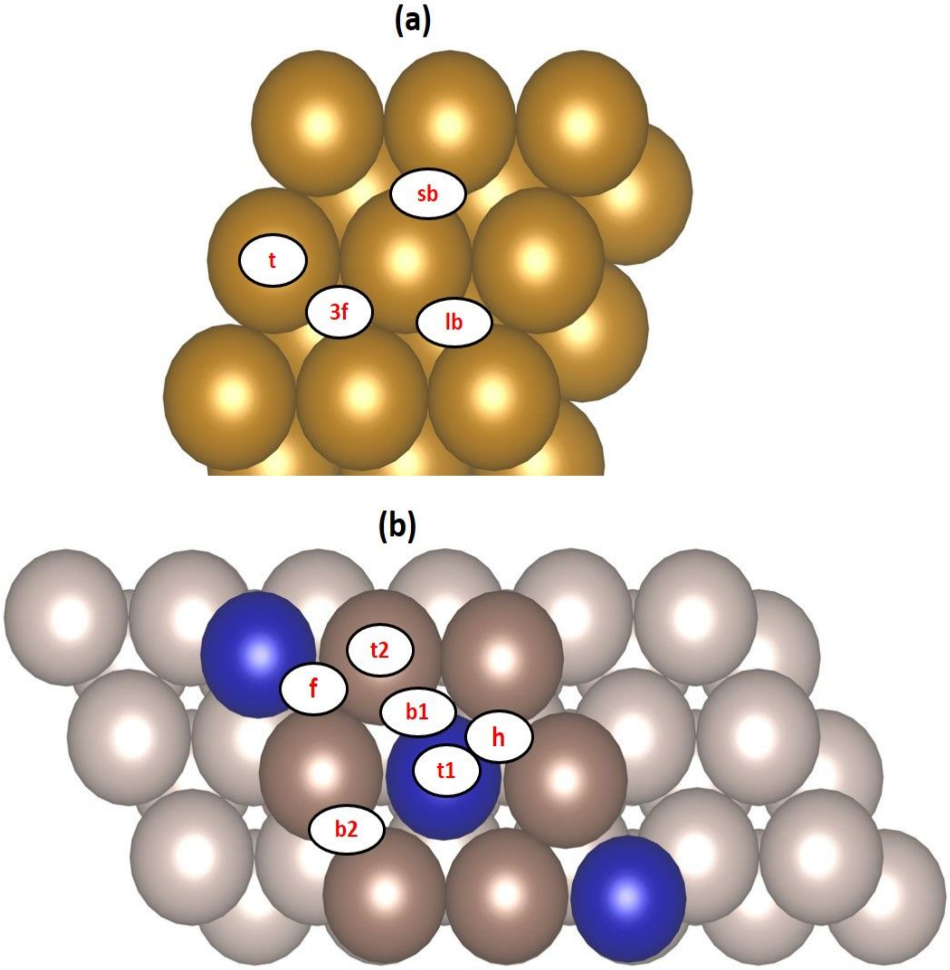

Figure S1: The top view of the various adsorption positions on a) pure Fe(110) and b) Co/stepped Ru(0001) surface having P1 pattern (t1: top 1, t2: top 2, b1: 2-fold bridge 1, b2: 2-fold bridge 2, h: hcp hollow, f: fcc hollow). Ru: dark brown (topmost layer); Ru: light brown (2nd,3rd and 4th layer of the stepped surface); Co: dark blue; Fe: brown.

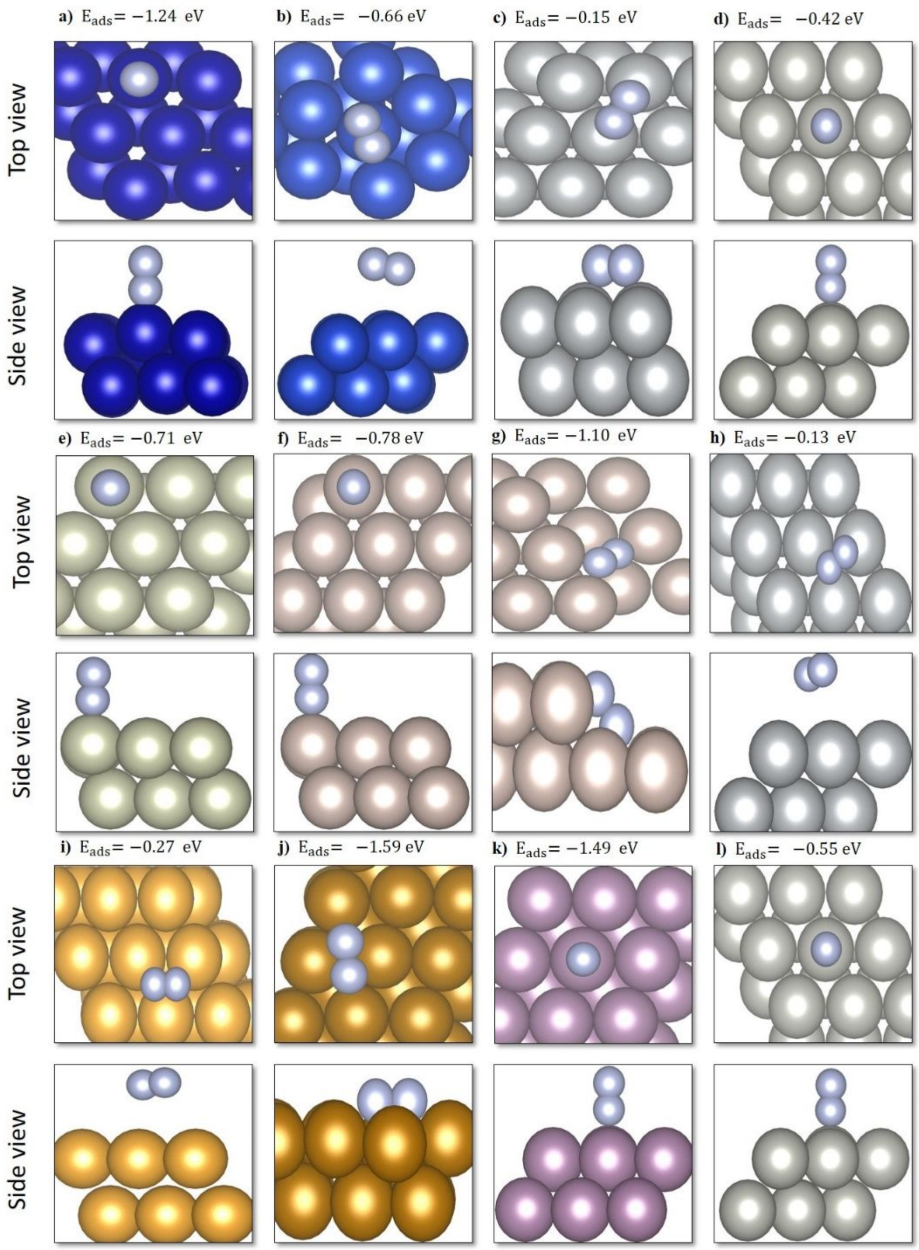

Figure S2: Top and side views of adsorbed N2 molecule on a) Co, b) Cu, c) Ni, d) Pd, e) Rh, f) Ru, g) stepped Ru, h) and Ag; i) Au, j) Fe, k) Mo and l) Pt surfaces; (N: light blue , Co: dark blue; Cu: blue; Ni: light gray; Pd: gray; Rh: Gold; Ru: light purple; Ag: dark gray, Au: yellow; Fe: brown; Mo: purple and Pt: gray).

Table S1: Structural parameters and adsorption energy (E_{ads}) of the most favorable N_2 adsorption configuration on various mono-metallic surfaces.

Surface	Parameter		
	$d_{(N-\mathbf{N})}$ (Å)	$d_{(surf-ads)}$ * (Å)	E_{ads} (eV)
**Gas** [illegible]	1.10998	-	-
**Ag(111)**	1.11	3.67	-0.13
**Au(111)**	1.11	3.77	-0.27
**Co(0001)**	1.13	1.78	-1.24
**Cu(111)**	1.11	3.22	-0.66
**Mo(110)**	1.13	2.08	-1.49
**Fe(110)**	1.29	1.99	-1.59
**Ni(111)**	1.17	1.93	-0.15
**Pd(111)**	1.12	2.02	-0.42
**Pt(111)**	1.12	1.95	-0.55
**Rh(111)**	1.13	1.94	-0.71
**Ru(0001)**	1.13	1.97	-0.78
**Stepped** [illegible]	1.32	2.03/2.08 [illegible]	-1.09

Table S2: The adsorption energies of N_2 at different adsorption sites on pure metal surfaces.

**Metal**	**structure**	**Adsorption site**	E_{ads} **(eV)**
Ag(111)	fcc	top	-0.12
		hcp	-0.127
		fcc	-0.130
		2f	-0.129
Au(111)	fcc	top	-0.272
		hcp	-0.150
		fcc	-0.154
		2f	-0.150
Co(0001)	hcp	top	-0.384
		hcp	-0.432

		fcc	-0.486
		2f	-1.243
Cu(111)	fcc	top	-0.29
		hcp	-0.662
		fcc	-0.374
		2f	-0.295
Ni(111)	fcc	top	-0.129
		hcp	-0.152
		fcc	-0.153
		2f	-0.152
Pd(111)	fcc	top	-0.169
		hcp	-0.422
		fcc	-0.420
		2f	-0.216
Pt(111)	fcc	top	-0.166
		hcp	-0.550
		fcc	-0.545
		2f	-0.183
Fe(110)	bcc	top	-1.09
		sb	-1.59
		lb	-1.29
		3f	-1.59
Mo(110)	bcc	top	-1.488
		sb	-3.792
		lb	-1.490
		3f	-3.912 (N2 dissociated spontaneously)
Ru(0001)	hcp	top	-0.774
		hcp	0.129
		fcc	-0.776
		2f	-0.775
Stepped Ru(0001)	hcp	Bridge (step-terrace)	-1.095
		fcc at step	-1.031
		hcp at step	-0.602
		hcp at terrace	-0.549

Rh(111)	fcc	top	-0.705
		hcp	-0.226
		fcc	-0.226
		2f	-0.225

Table S3: The adsorption energies of atomic N at different adsorption sites on pure metal surfaces.

**Metal**	**structure**	**Adsorption site**	$\mathbf{E_{ads}}$ **(eV)**
Ag(111)	fcc	top	2.48
		hcp	2.48
		fcc	2.40
		2f	2.40
Au(111)	fcc	top	2.81
		hcp	2.92
		fcc	2.81
		2f	2.10
Co(0001)	hcp	top	-0.73
		hcp	-0.80
		fcc	-0.79
		2f	-0.75
Cu(111)	fcc	top	1.03
		hcp	1.06
		fcc	1.03
		2f	1.25
Ni(111)	fcc	top	-0.18
		hcp	-0.18
		fcc	-0.24
		2f	-0.18
Pd(111)	fcc	top	0.42
		hcp	0.42
		fcc	0.40
		2f	0.40
Pt(111)	fcc	top	0.15
		hcp	0.34

		fcc	0.15
		2f	0.15
Fe(110)	bcc	top	-1.95
		sb	-1.95
		lb	-1.61
		3f	-1.95
Mo(110)	bcc	top	-2.04
		sb	-2.04
		lb	-2.01
		3f	-2.04
Ru(0001)	hcp	top	-0.90
		hcp	-0.90
		fcc	-0.27
		2f	-0.90
Stepped Ru(0001)	hcp	Bridge (step-terrace)	-0.79
		fcc at step	-0.24
		hcp at step	-0.90
		hcp at terrace	-0.68
Rh(111)	fcc	top	-0.48
		hcp	-0.61
		fcc	-0.48
		2f	-0.48

Table S4: Total energies of bare Mo-based alloy surfaces for various patterns.

**Alloy**	**Patterns**		
	**P3**	**P4**	**P5**
**Ag/ Mo(110)**	-16347.38	-16347.29	-16347.32
**Au/Mo(110)**	-17607.30	-17607.23	-17607.26
**Co/Mo(110)**	-16402.70	-16402.67	-16402.67
**Cu/Mo(110)**	-16730.60	-16730.57	-16730.53
**Fe/Mo(110)**	-16269.34	-16269.30	-16269.32
**Ni/Mo(110)**	-15838.91	-15838.84	-15838.87
**Pd/Mo(110)**	-16275.12	-16275.00	-16275.06
**Pt/Mo(110)**	-17523.90	-17523.81	-17523.85

**Rh/Mo(110)**	-16691.40	-16691.34	-16691.39
**Ru/Mo(110)**	-16576.36	-16576.36	-16576.38

Table S5: Total energies of bare Ru-based alloy surfaces for various patterns.

**Alloy**	**Patterns**	
	**P1**	**P2**
**Ag /st.Ru(0001)**	-26956.45	-26956.46
**Au /st.Ru(0001)**	-28216.31	-28216.34
**Co /st.Ru(0001)**	-27011.76	-27011.76
**Cu /st.Ru(0001)**	-27339.75	-27339.75
**Fe /st.Ru(0001)**	-26878.51	-26878.52
**Ir /st.Ru(0001)**	-28055.47	-28055.46
**Mo /st.Ru(0001)**	-26982.37	-26982.36
**Ni /st.Ru(0001)**	-26447.95	-26447.95
**Pd /st.Ru(0001)**	-26884.09	-26884.11
**Pt /st.Ru(0001)**	-28132.85	-28132.85
**Rh /st.Ru(0001)**	-27300.39	-27300.39

Table S6: The adsorption energies of N_2 molecule at different adsorption sites on Mo-based alloy surfaces.

**Surface**	**Site orption**	E_{ads} **(eV)**	**Surface**	**Site orption**	E_{ads} **(eV)**	**Surface**	**Site orption**	E_{ads} **(eV)**
**Ag/Mo(110)**	h	-1.12	**Co/Mo(110)**	h	-1.341	**Fe/Mo(110)**	h	-1.27
	hh	-0.821		hh	-0.762		hh	-0.51
	lb	-0.781		lb	-1.141		lb	-1.43
	sb	-0.820		sb	-1.214		sb	-1.01
	t1	-0.816		t1	-0.854		t1	-0.84
	t2	-0.101		t2	-1.080		t2	-0.62
**Au/Mo(110)**	h	-1.126	**Cu/Mo(110)**	h	-1.57	**Ni/Mo(110)**	h	-1.267
	hh	-0.767		hh	-0.886		hh	-0.750
	lb	-0.782		lb	-1.242		lb	-0.829
	sb	-0.821		sb	-1.177		sb	-0.831
	t1	-0.821		t1	-1.146		t1	-0.884
	t2	-0.146		t2	-0.458		t2	-0.643

**Pd/Mo(110)**	h	-1.127	**Pt/Mo(110)**	h	-1.123	**Ru/Mo(110)**	h	-1.613
	hh	-0.827		hh	-0.80		hh	-0.96
	lb	-0.829		lb	-0.795		lb	-0.96
	sb	-0.827		sb	-0.826		sb	-1.61
	t1	-0.821		t1	-0.82		t1	-0.75
	t2	-0.217		t2	-0.10		t2	-0.87
**Rh/Mo(110)**	h	-1.271						
	hh	-0.975						
	lb	-0.974						
	sb	-0.448						
	t1	-0.912						
	t2	-0.709						

Table S7: The adsorption energies of N_2 molecule at different adsorption sites on Fe-based alloy surfaces.

**Surface**	**Site orption [illegible]**	E_{ads} **(eV)**	**Surface**	**Site orption [illegible]**	E_{ads} **(eV)**	**Surface**	**Site orption [illegible]**	E_{ads} **(eV)**
**Ag/Fe(110)**	h	-0.691	**Co/Fe(110)**	h	-1.5746	**Mo/Fe(110)**	h	-1.669
	hh	-0.730		hh	-1.383		hh	-1.9759
	lb	-0.394		lb	-1.147		lb	-1.9756
	sb	-0.693		sb	-1.458		sb	-1.889
	t1	-0.729		t1	-1.035		t1	-1.195
	t2	-0.729		t2	-1.111		t2	-1.372
**Au/Fe(110)**	h	-0.5457	**Cu/Fe(110)**	h	-1.586	**Ni/Fe(110)**	h	-[illegible]
	hh	-0.507		hh	-0.999		hh	-0.895
	lb	-0.419		lb	-1.023		lb	-0.540
	sb	-0.5466		sb	-1.023		sb	-[illegible]
	t1	-0.508		t1	-1.023		t1	-1.066
	t2	-0.485		t2	-0.551		t2	-0.962
**Pd/Fe(110)**	h	-0.915	**Pt/Fe(110)**	h	-1.101	**Ru/Fe(1[illegible]**	h	-1.917
	hh	-1.021		hh	-1.051		hh	-1.467
	lb	-0.647		lb	-0.409		lb	-1.372
	sb	-0.914		sb	-1.1021		sb	-1.797

	t1	-1.019	t1	-1.050	t1	-1.413
	t2	-0.645	t2	-0.598	t2	-1.357
**Rh/Fe(110)**	h	-1.198				
	hh	-0.927				
	lb	-0.807				
	sb	-1.201				
	t1	-0.973				
	t2	-1.066				

N_2 Dissociation on Pure Metals. The $N \equiv N$ bond scission often controls the overall reaction rate of ammonia synthesis. Here, the elementary step of N_2 dissociation to atomic N on mono-metals (reference surfaces) and binary metal alloy surfaces are investigated in order to unveil the effect of the TM alloying on the activation barrier for $N \equiv N$ bond cleavage.

Upon N_2 adsorption on various surfaces studied, the $N \equiv N$ bond length was increased, which indicate that this triple bond is weakened and that nitrogen molecule might dissociate under these adsorption modes. Therefore, the N_2 decoupling process was investigated using the most stable adsorption configuration as the initial state. The initial state (IS), transition state (TS) and final state (FS) for the reaction pathway for N–N decoupling in the N_2 molecule on all monometallic surfaces are depicted in **Figure S3 and S4** (Supporting Information). In the cases of Ag(111) and Au(111) surfaces, the N_2 attached to the 3-fold hollow site is directly dissociated through the TS state. Subsequently, the two N atoms gradually diffuse to the two neighboring hollow sites, thus forming the FS. A similar dissociation pathway was noticed on Co(0001), Mo(110), Pd(111), Pt(111), Rh(111) and Ru(0001) surfaces, however, the N_2 molecule, on the previous surfaces, possesses perpendicular orientation, is bonded to the top site in the IS state. For the TS structure of N_2 adsorption on these monometallic surfaces, the $N \equiv N$ bond is completely cleaved and the distances between the two N atoms ($d_{(N-N)}$) are 2.23 Å (Ag), 2.15 Å (Cu), 1.90 Å (Pt), 1.88 Å (Pd), 1.80 Å (st. Ru), 1.75 Å (Rh), 1.74 Å (Ni), 1.74 Å (Au), 1.60 Å (Ru), 1.59 Å (Co), 1.36 Å (Mo) and 1.21 Å (Fe). As expected, owing to the strong interatomic bond of the adsorbate, the process is unaffordable on Ag(111), Cu(111), Pd(111), Pt(111), Rh(111), Ru(0001) and Ni(111), surmounting for high energy barriers of 5.95, 3.59, 3.4, 3.32, 2.09, 1.78 and 1.62 eV respectively. Moreover, the process is highly endothermic on these surfaces, with exception of Rh(111) (-0.07 eV), Ru(0001) (-0.74 eV) and Ni(111) (-0.03 eV), requiring reaction energies between 1.39-5.44 eV. No activation barrier was obtained for N_2 dissociation on Au(111). The computed kinetic barrier on flat Ru(0001) surface (1.78 eV) is in good agreement with the values 1.8 and 1.9 eV

reported in the literature.[4,8] Moreover, molecular beam scattering measurements indicated an activation energy range of 1 – 2 eV for the N_2 bond cleavage.[9] Similar dissociation pathway (IS,TS and FS structures) on Ru(0001) terrace was revealed by Mortensen et al.[10]. On the other hand, lower activation energies for $N \equiv N$ bond dissociation are required on Fe(110), st. Ru(0001), Mo(110) and Co(0001), accounting for 0.45, 0.59, 0.64 and 0.78 eV, respectively. Interestingly, the dissociation process is thermodynamically preferable over Mo(110) and Fe(110) surfaces, possessing high exothermic character of -3.04 and -2.09 eV, respectively. The kinetic barrier dissociation on Fe(110) agrees well with the experimental value of 27 KJ/mol (0.28 eV) measured by Egeberg et al.[11]. As expected, the *dissociation barrier is reduced by 1.19 eV on stepped surface as compared to that on planar counterpart*. Similarly, Dahl and co-workers [4] reported that the activation energy for N_2 dissociation is more than 1 eV lower on the Ru(0001) step edge than on the terrace surface. By comparison of TS configuration of structure (e) and (f) in **Figure S4** (Supporting Information), it can be seen that they have crucial similarities. In both structures, atomic N is located on the most favorable 3-fold hollow hcp site, whereas the other N is attached to bridge site. The N_2 molecule strongly prefers the step site because both nitrogen atoms will not have to share any Ru atoms as nearest neighbors. The variation in activation energy is a result of the fact that five Ru surface atoms are associated with TS structure at the step site rather than four on the terrace. Accordingly, the TS geometry at the step site avoids indirect repulsive interactions that are responsible for high activation energy on the terrace.[4] Moreover, the step atoms possess high reactivity than the terrace atoms, which gives rise to additional lowering of the activation energy. Based on the activation energy (E_a), Fe(110), st. Ru(0001) and Mo(110) presented the highest activity and expected to be potent catalysts for ammonia synthesis, thereby the bimetallic alloys of these metals were also considered for investigating the N_2 dissociation.

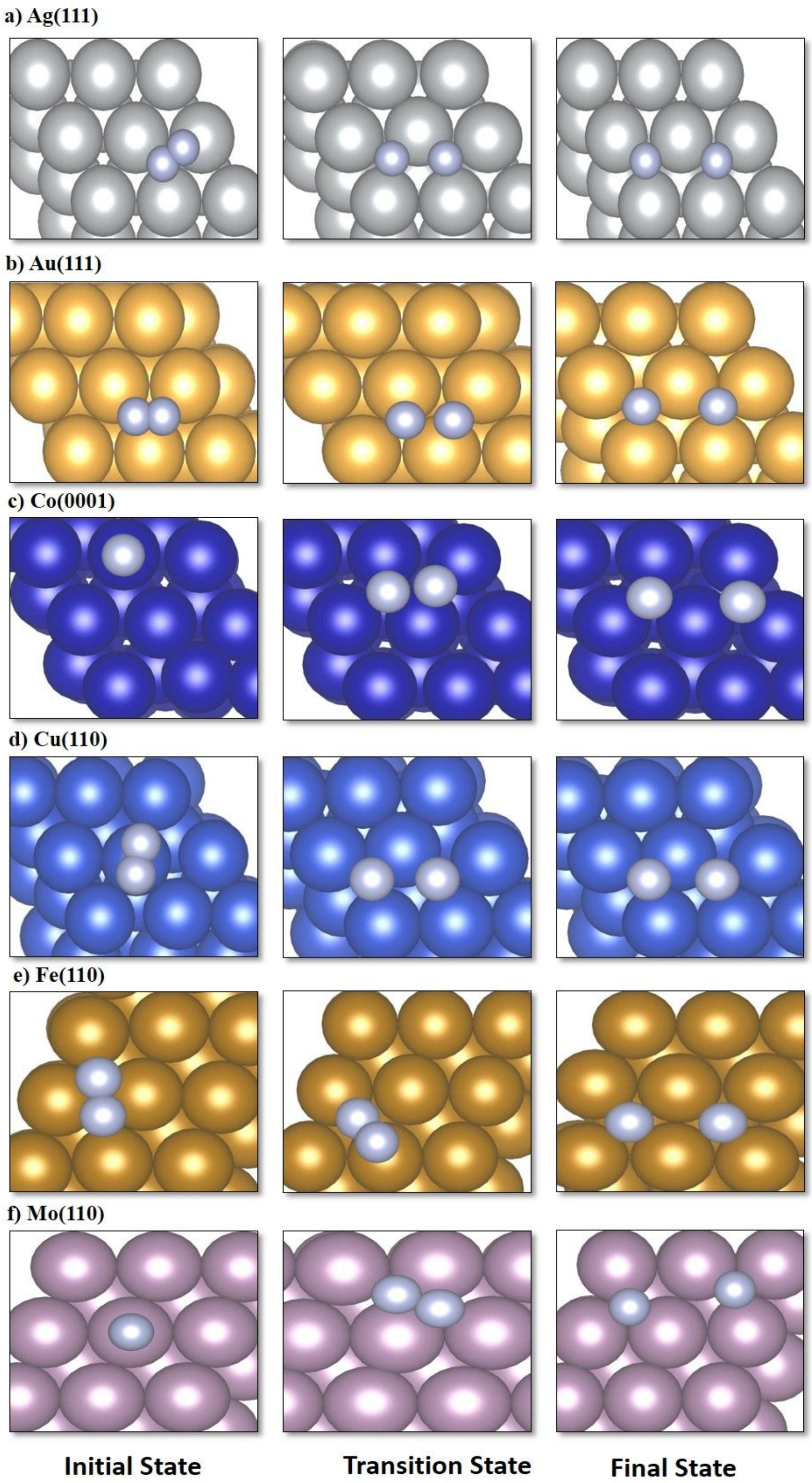

Figure S3: Top views of initial , transition and final states of N_2 dissociation on a) Ag(111), b) Au(111), c) Co(0001), d) Cu(111), e) Fe(110) and f) Mo(110) surfaces.

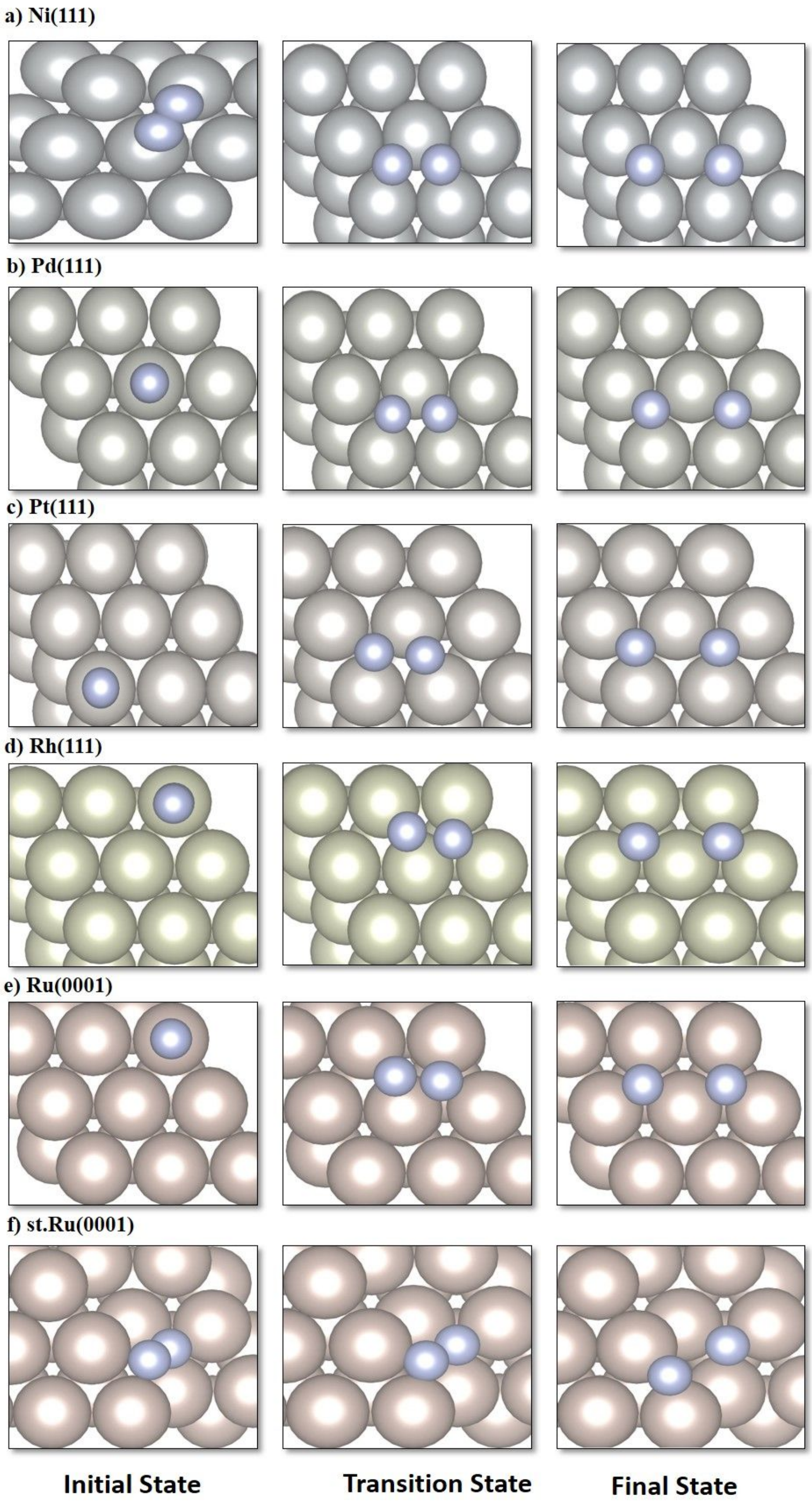
a) Ni(111)
b) Pd(111)
c) Pt(111)
d) Rh(111)
e) Ru(0001)
f) st.Ru(0001)
Initial State
Transition State
Final State

Figure S4: Top views of initial , transition and final states of N_2 dissociation on a) Ni(111), b) Pd(111), c) Pt(111), d) Rh(111), e) Ru(0001) and f) st.Ru(0001) surfaces.

Table S8: Effect of alloying (chemical strain) on lattice parameters for the various TM/Mo(110) surfaces.

Surface	a %	b %
**Ag-Mo**	0.07	0.60
**Au-Mo**	0.43	0.87
**Co-Mo**	3.30	3.56
**Cu-Mo**	4.22	3.84
**Fe-Mo**	0.03	0.25
**Ni-Mo**	0.12	0.08
**Pd-Mo**	0.38	0.53
**Pt-Mo**	0.55	0.55
**Rh-Mo**	0.17	0.44
**Ru-Mo**	0.16	0.16

Table S9: Effect of alloying (chemical strain) on lattice parameters of various TM/Fe(110) surfaces.

Surface	a %	b %
**Ag-Fe**	0.28	0.28
**Au-Fe**	0.19	0.19
**Co-Fe**	0.27	0.27
**Cu-Fe**	0.36	0.07
**Mo-Fe**	0.58	0.54
**Ni-Fe**	0.16	0.16
**Pd-Fe**	0.90	0.90
**Pt-Fe**	0.98	0.98
**Rh-Fe**	0.56	0.56

**Ru-Fe**	0.38	0.38

Table S10: Effect of alloying (chemical strain) on lattice parameters of various TM/st.Ru(0001) surfaces.

Surface	a %	b %
**Ag-Ru**	0.01	0.02
**Au-Ru**	0.08	0.07
**Co-Ru**	6.50	6.70
**Cu-Ru**	2.18	2.21
**Fe-Ru**	0.14	0.07
**Mo-Ru**	0.07	0.09
**Ni-Ru**	0.07	0.05
**Pd-Ru**	0.10	0.13
**Pt-Ru**	0.12	0.16
**Rh-Ru**	0.13	0.12

Table S11: The adsorption energies of atomic N at different adsorption sites on TM/st.Ru(0001) surfaces.

Surface	E_{ads} (eV)
**Ag-Ru**	-0.15
**Au-Ru**	0.10
**Co-Ru**	-0.87
**Cu-Ru**	-0.55
**Fe-Ru**	-0.59
**Mo-Ru**	-1.55
**Ni-Ru**	-0.77
**Pd-Ru**	-0.28
**Pt-Ru**	-0.21
**Rh-Ru**	-0.53

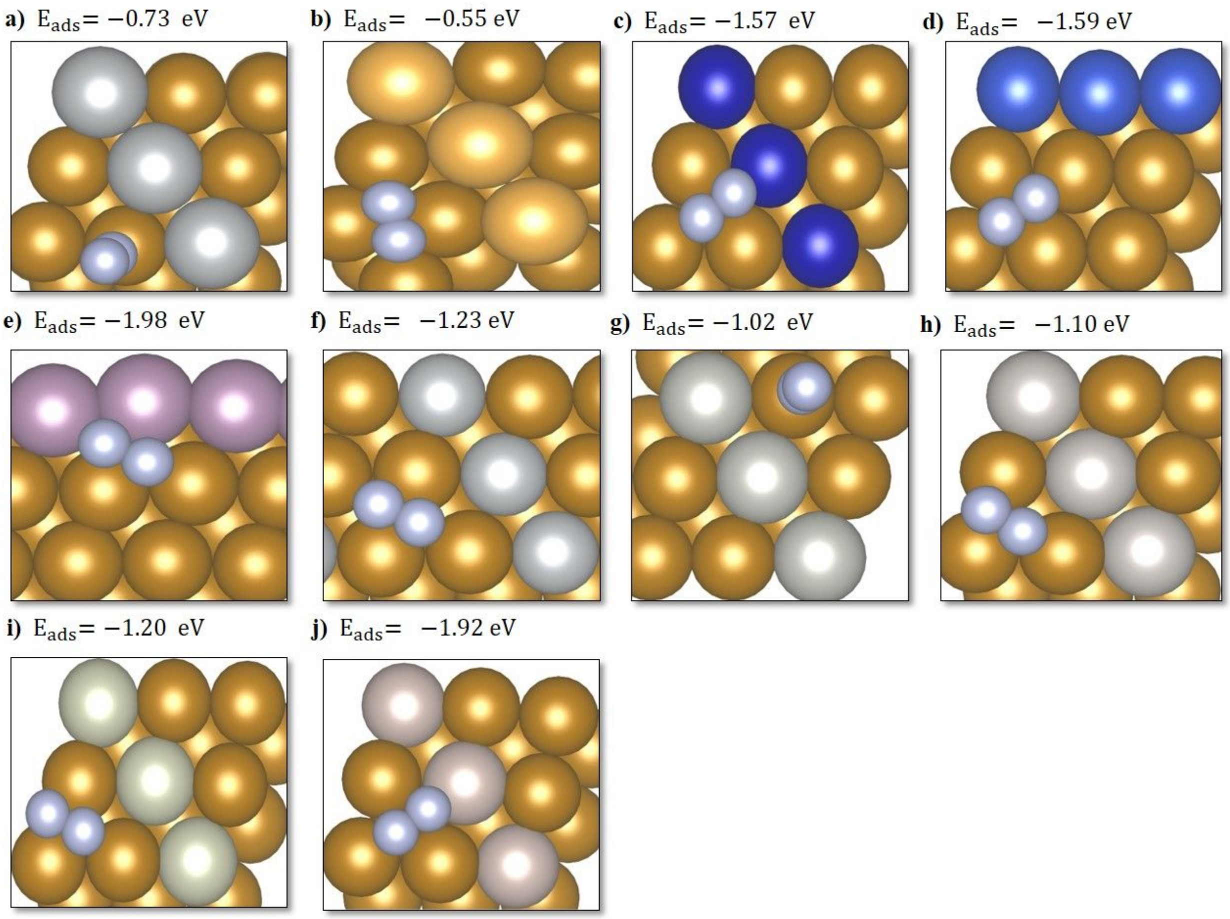

Figure S5: Top views of adsorbed N_2 molecule on **Fe-based alloy** surfaces: **a)** Ag/Fe(110); **b)** Au/Fe(110); **c)** Co/Fe(110); **d)** Cu/Fe(110); **e)** Mo/Fe(110); **f)** Ni/Fe(110); **g)** Pd/Fe(110); **h)** Pt/Fe(110); **i)** Rh/Fe(110); **j)** Ru/Fe(110). (N: light blue; Ag: light gray; Au: yellow, Co: dark blue; Cu: blue; Mo: purple; Fe: brown; Ni: light gray; Pd: dark gray; Pt: gray; Rh: Gold; Ru: light brown).

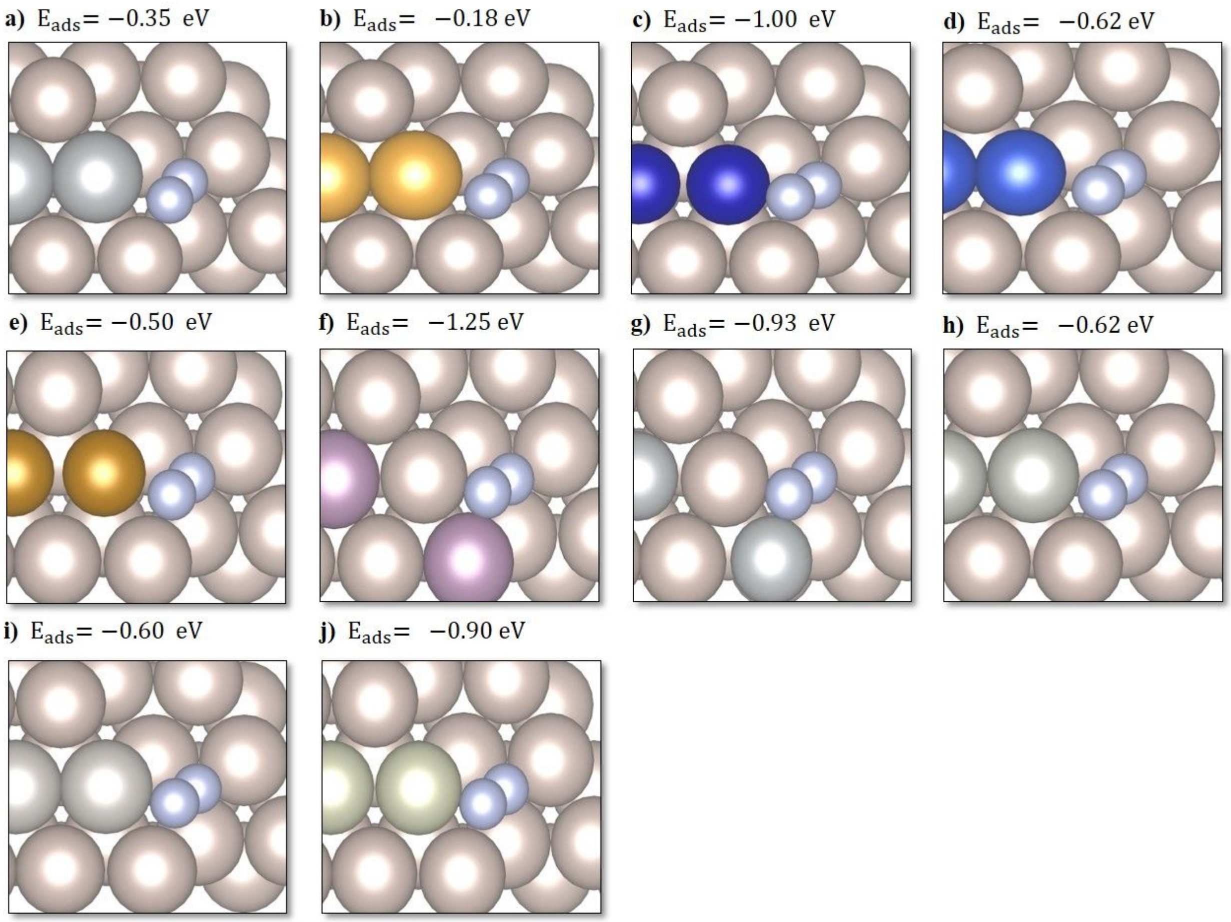

Figure S6: Top views of adsorbed N_2 molecule on **Ru-based alloy** surfaces: **a)** Ag/st.Ru(0001); **b)** Au/st.Ru(0001); **c)** Co/st.Ru(0001); **d)** Cu/st.Ru(0001); **e)** Fe/st.Ru(0001); f) Mo/st.Ru(0001); **g)** Ni/st.Ru(0001); **h)** Pd/st.Ru(0001); **i)** Pt/st.Ru(0001); **j)** Rh/st.Ru(0001). (N: light blue; Ag: light gray; Au: yellow, Co: dark blue; Cu: blue; Mo: purple; Fe: brown; Ni: light gray; Pd: dark gray; Pt: gray; Rh: Gold; Ru: light brown).

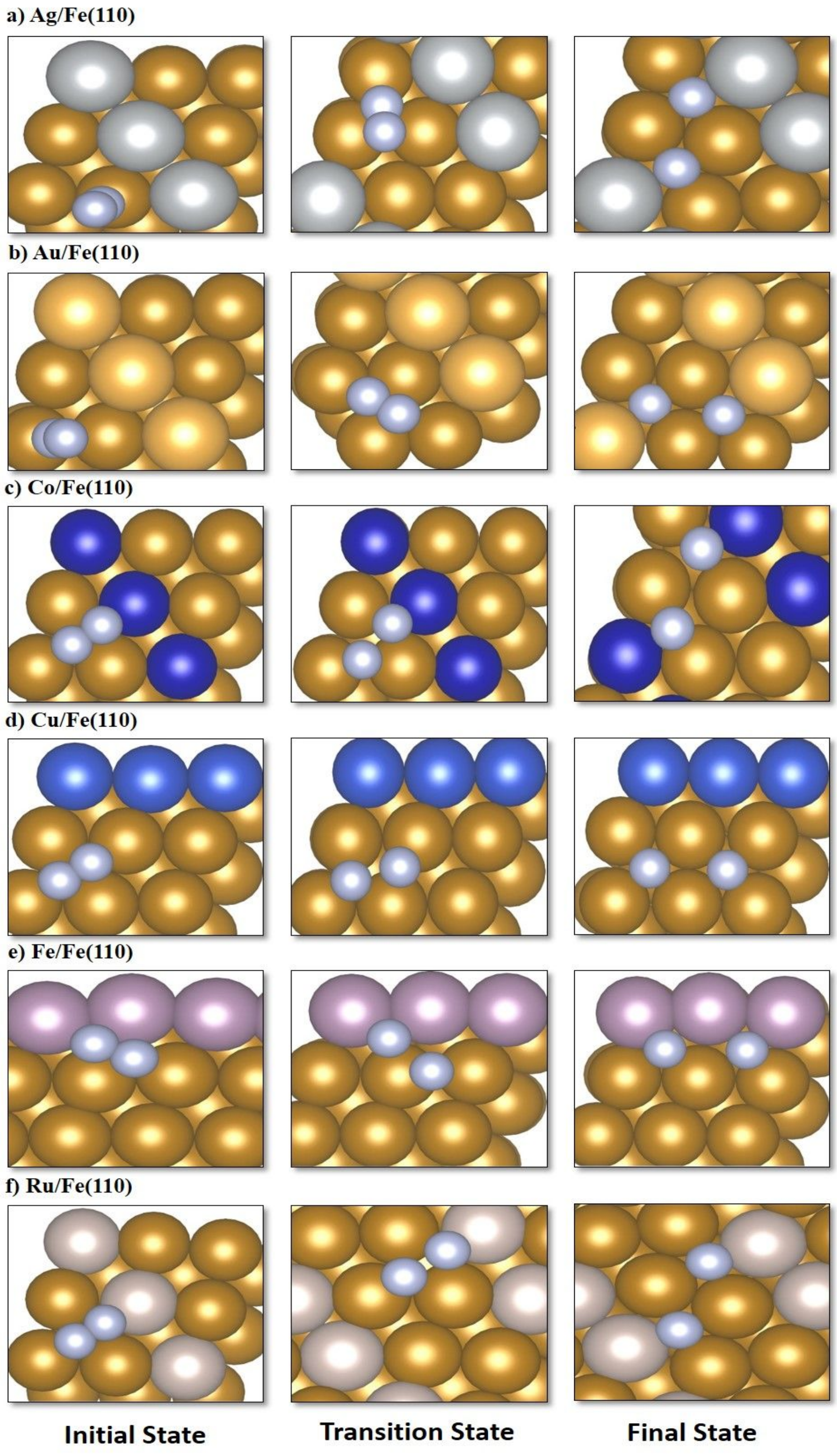

Figure S7: Top views of initial state (IS), transition state (TS) and final state (FS) of N_2 dissociation on **Fe-based alloys**: **a)** Ag/Fe(110); **b)** Au/Fe(110); **c)** Co/Fe(110); **d)** Cu/Fe(110); **e)** Mo/Fe(110); and **f)** Ru/Fe(110) surfaces.

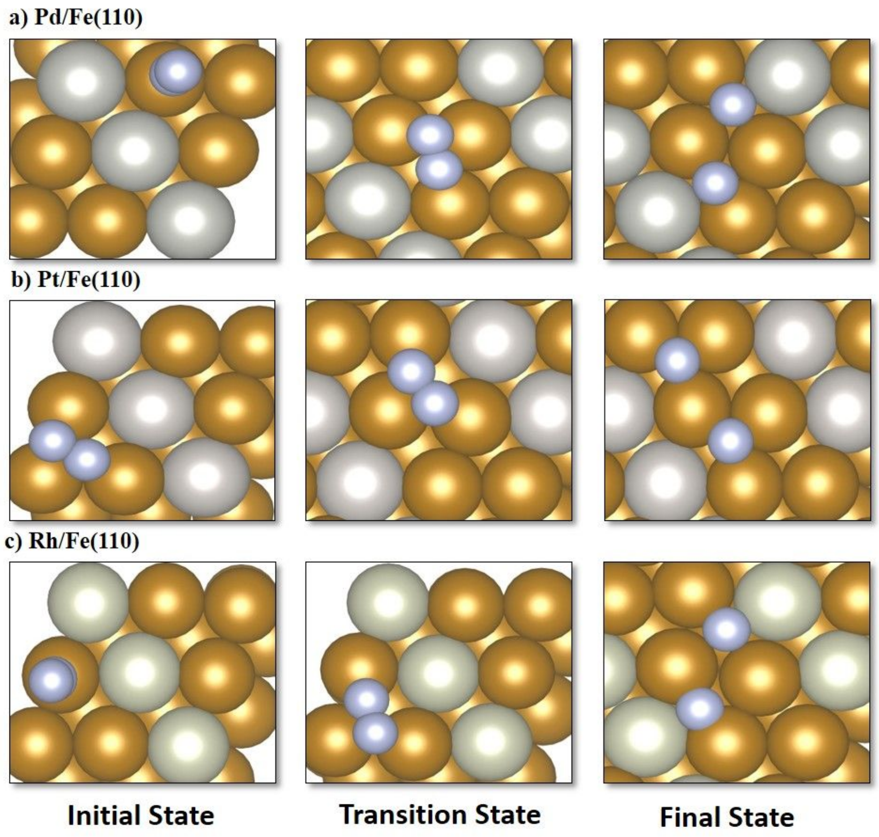

Figure S8: Top views of initial (IS), transition (TS) and final states (FS) of N_2 dissociation on **Fe-based alloys**: **a)** Pd/ Fe(110); **b)** Pt/ Fe(110); and **c)** Rh/Fe(110) surfaces.

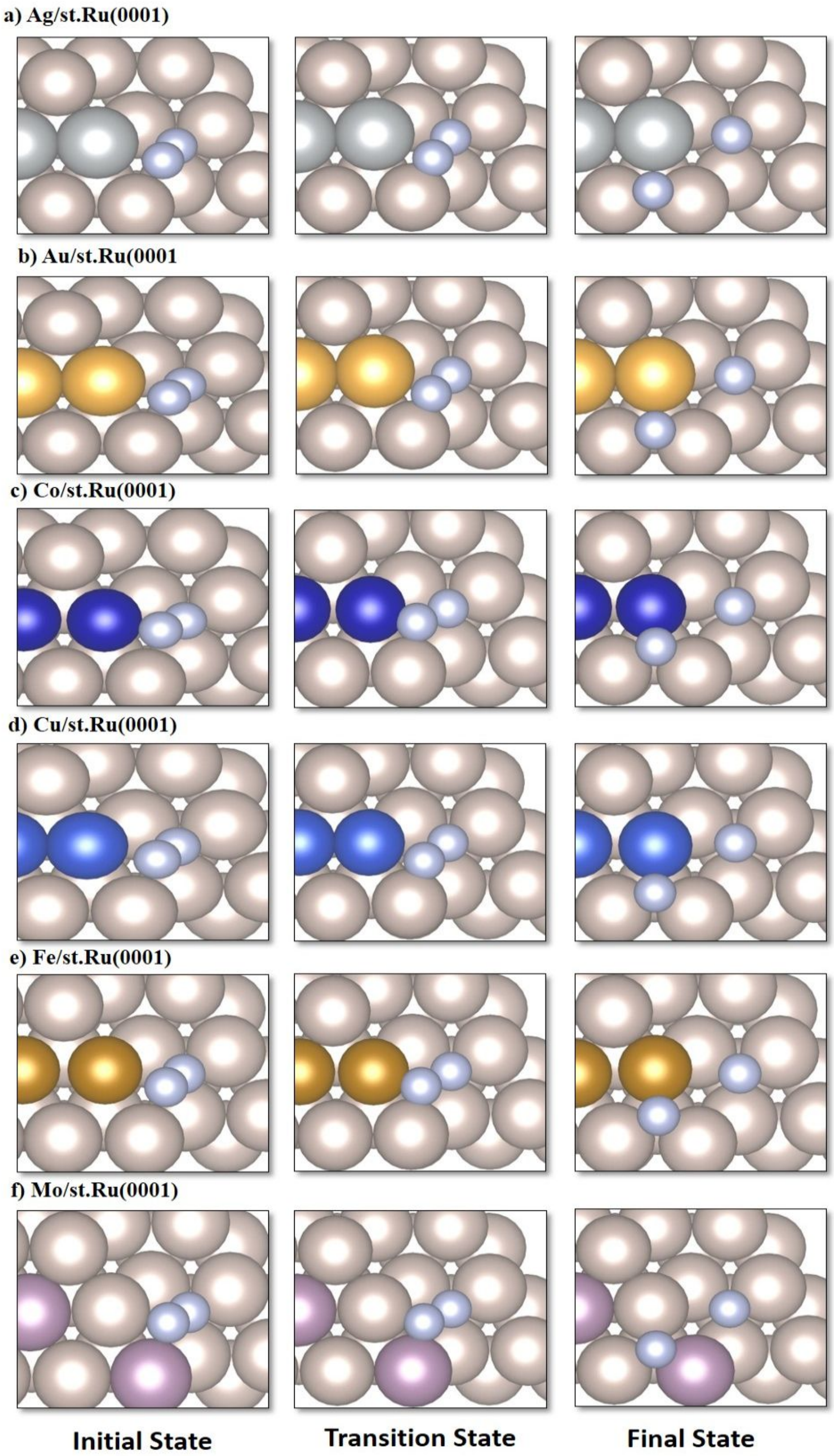

Figure S9: Top views of initial state (IS), transition state (TS) and final state (FS) of N_2 dissociation on **st.Ru-based alloys**: **a)** Ag/st.Ru(0001); **b)** Au/st.Ru(0001); **c)** Co/st.Ru(0001); **d)** Cu/st.Ru(0001); **e)** Fe/st.Ru(0001); and f) Mo/st.Ru(0001) surfaces.

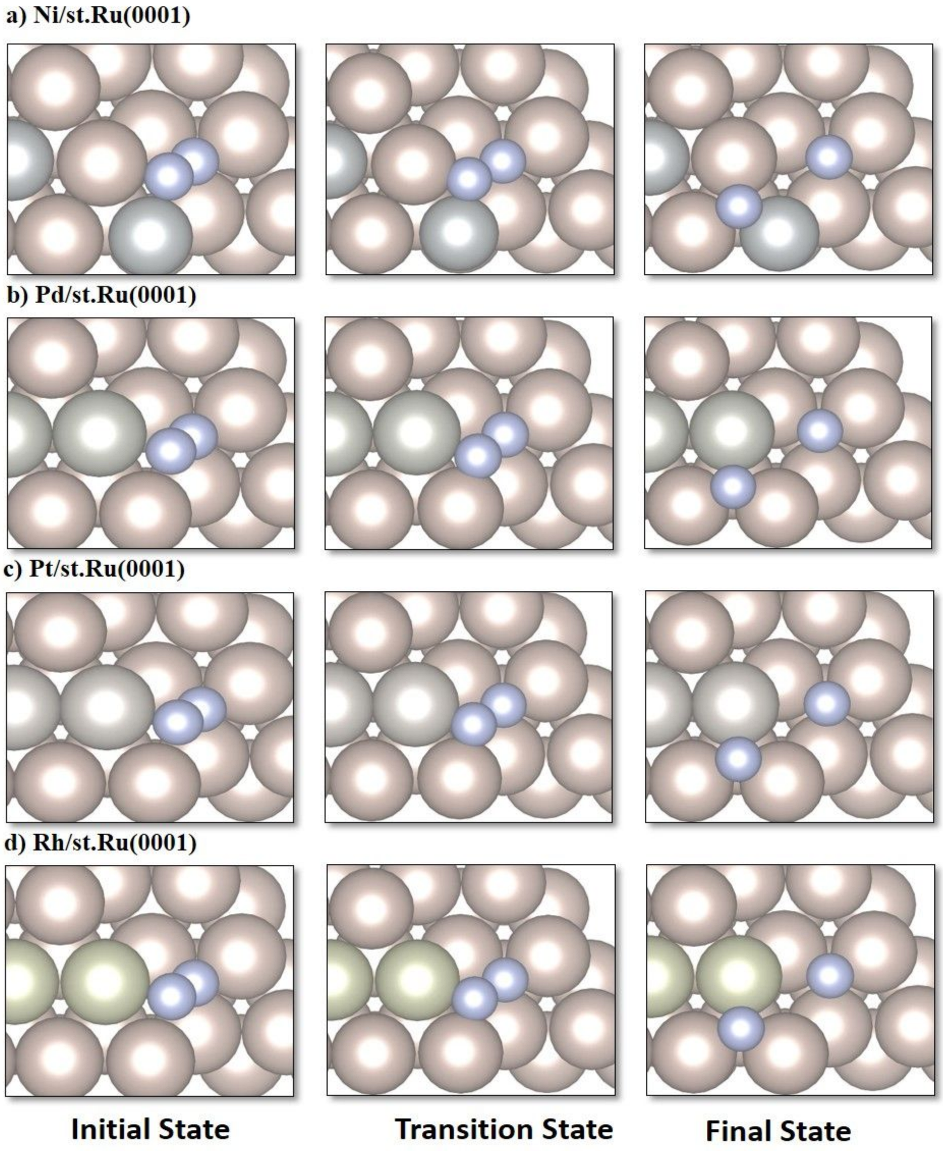

Figure S10: Top views of initial state (IS), transition state (TS) and final state (FS) of N_2 dissociation on **st.Ru-based alloys**: **a)** Ni/st.Ru(0001); **b)** Pd/st.Ru(0001); **c)** Pt/st.Ru(0001); and **d)** Rh/st.Ru(0001) surfaces.

References:

(1) Tafreshi, S. S.; Roldan, A.; de Leeuw, N. H. Density Functional Theory Calculations of the Hydrazine Decomposition Mechanism on the Planar and Stepped Cu(111) Surfaces. *Phys. Chem. Chem. Phys.* **2015**, *17* (33), 21533–21546. https://doi.org/10.1039/C5CP03204K.

(2) Lykke, K. R.; Kay, B. D. Rotational Rainbows in the Inelastic Scattering of N2 from Au(111). *Journal of Physics: Condensed Matter* **1991**, *3* (S), S65–S70. https://doi.org/10.1088/0953-8984/3/s/010.

(3) Martirez, J. M. P.; Carter, E. A. Thermodynamic Constraints in Using AuM (M = Fe, Co, Ni, and Mo) Alloys as N2 Dissociation Catalysts: Functionalizing a Plasmon-Active Metal. *ACS Nano* **2016**, *10* (2), 2940–2949. https://doi.org/10.1021/acsnano.6b00085.

(4) Dahl, S.; Logadottir, A.; Egeberg, R. C.; Larsen, J. H.; Chorkendorff, I.; Törnqvist, E.; N\orskov, J. K. Role of Steps in N2 Activation on Ru(0001). *Phys. Rev. Lett.* **1999**, *83* (9), 1814–1817. https://doi.org/10.1103/PhysRevLett.83.1814.

(5) Morgan, G. A.; Sorescu, D. C.; Kim, Y. K.; Yates, J. T. Comparison of the Adsorption of N2 on Ru(109) and Ru(001) – A Detailed Look at the Role of Atomic Step and Terrace Sites. *Surf Sci* **2007**, *601* (17), 3533–3547. https://doi.org/https://doi.org/10.1016/j.susc.2007.06.019.

(6) Zambelli, T.; Wintterlin, J.; Trost, J.; Ertl, G. Identification of the "Active Sites" of a Surface-Catalyzed Reaction. *Science (1979)* **1996**, *273* (5282), 1688–1690. https://doi.org/10.1126/science.273.5282.1688.

(7) Jacobi, K. Nitrogen on Ruthenium Single-Crystal Surfaces. *physica status solidi (a)* **2000**, *177* (1), 37–51. https://doi.org/https://doi.org/10.1002/(SICI)1521-396X(200001)177:1<37::AID-PSSA37>3.0.CO;2-Y.

(8) Kedalo, Y. M.; Knizhnik, A. A.; Potapkin, B. V. Theoretical Analysis of Energy Efficiency of Plasma-Assisted Heterogeneous Activation of Nitrogen for Ammonia Synthesis. *Plasma Chemistry and Plasma Processing* **2021**, *41* (5), 1279–1291. https://doi.org/10.1007/s11090-021-10199-y.

(9) Romm, L.; Katz, G.; Kosloff, R.; Asscher, M. Dissociative Chemisorption of N2 on Ru(001) Enhanced by Vibrational and Kinetic Energy: Molecular Beam Experiments and Quantum Mechanical Calculations. *J Phys Chem B* **1997**, *101* (12), 2213–2217. https://doi.org/10.1021/jp962599o.

(10) Mortensen, J. J.; Hammer, B.; N\orskov, J. K. Alkali Promotion of N2 Dissociation over Ru(0001). *Phys. Rev. Lett.* **1998**, *80* (19), 4333–4336. https://doi.org/10.1103/PhysRevLett.80.4333.

(11) Egeberg, R. C.; Dahl, S.; Logadottir, A.; Larsen, J. H.; Nørskov, J. K.; Chorkendorff, I. N2 Dissociation on Fe(110) and Fe/Ru(0001): What Is the Role of Steps? *Surf Sci* **2001**, *491* (1), 183–194. https://doi.org/https://doi.org/10.1016/S0039-6028(01)01397-8.
